# Ultrasound‐Triggered Gelation for Restoring Biomechanical Properties of Degenerated Functional Spinal Units

**DOI:** 10.1002/adhm.202501823

**Published:** 2025-12-10

**Authors:** Veerle A. Brans, Anna P. Constantinou, Matthew J. Kibble, Valeria Nele, Daniel Reumann, Luca Bau, Sebastien J. P. Callens, James P. K. Armstrong, Nicolas Newell, Constantin C. Coussios, Molly M. Stevens, Michael D. Gray

**Affiliations:** ^1^ Institute of Biomedical Engineering University of Oxford Oxford OX3 7LD UK; ^2^ Department of Materials Department of Bioengineering Institute of Biomedical Engineering Imperial College London London SW7 2AZ UK; ^3^ Department of Physiology Anatomy and Genetics Department of Engineering Science Kavli Institute for Nanoscience Discovery University of Oxford Oxford OX1 3QU UK; ^4^ Department of Bioengineering White City Campus Sir Michael Uren Hub Imperial College London London W12 0BZ UK; ^5^ Present address: Department of Pharmacy University of Naples Federico II Via D. Montesano 49–80131 Naples Italy; ^6^ Present address: Orthopaedic Biomechanics Department of Biomedical Engineering Eindhoven University of Technology 5600 MB Eindhoven The Netherlands; ^7^ Present address: Department of Translational Health Sciences Bristol Medical School University of Bristol Bristol BS1 3NY UK

**Keywords:** biomechanics, hydrogel, intervertebral disc, spine, ultrasound

## Abstract

Lower back pain is closely associated with intervertebral disc (IVD) degeneration and is a leading cause of global disability. Existing treatment options are unable to provide suitable long‐term outcomes, and emerging strategies employing injectable biomaterials are hindered by factors including limited native tissue integration and depth‐ or time‐constrained gelation mechanisms. To overcome these issues, the present research evaluates a new concept employing ultrasound to remotely trigger in situ implant formation. The concept centers around an implant precursor biomaterial consisting of an anionic polysaccharide solution containing thermally sensitive liposomes loaded with ionic crosslinkers. Ultrasound‐mediated heating to 4–5 °C above normal body temperature triggers liposomal release of the crosslinking species, thereby initiating hydrogel formation. Optimization studies define the implant precursor material (1.5% wt/v sodium alginate seeded with calcium‐loaded liposomes (10–15 mm calcium chloride) and 6% wt/v glass microspheres) and the ultrasound parameters (0.95 MHz, 1.6 MPa amplitude, 87% duty cycle). Proof‐of‐concept experiments in degenerated ex vivo bovine IVDs indicate partial restoration of biomechanical function, with the implanted biomaterial well‐integrated into the disc tissue and without material herniation. These results offer promise for treating intervertebral disc degeneration, with continued refinement of biomaterials and protocols being essential for achieving robust in‐disc efficacy.

## Graphical abstract

This study introduces an innovative approach to treating intervertebral disc degeneration using ultrasound‐triggered in situ hydrogel formation. Proof‐of‐concept experiments using optimized biomaterial and ultrasound parameters demonstrate partial restoration of biomechanical function and successful integration into degenerated disc tissue, offering a promising avenue for advanced, minimally invasive therapies for lower back pain. Created in BioRender. Kaesbauer, S. (2025) https://BioRender.com/f99g314.

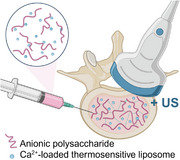

## 1 Introduction

Lower back pain ranks among the top ten causes of disability‐adjusted life years worldwide and is the primary cause of years lived with disability in high‐income countries. In 2020, an estimated 619 million people were affected by lower back pain worldwide, with projections indicating a 36.4% increase to 843 million by 2050.^[^ ^1^ ^]^ The degeneration of the intervertebral disc (IVD) accounts for ∼40% of cases of lower back pain and related radicular leg pain.^[^ ^2^, ^3^, ^4^ ^]^ The socioeconomic burden of lower back pain is estimated at £12 billion annually in the UK and ∼$85 billion in the USA,^[^ ^5^, ^6^ ^]^ and this cost is expected to increase due to the relationship between disc degeneration and factors such as aging and obesity.^[^ ^7^ ^]^

**Figure 1:**
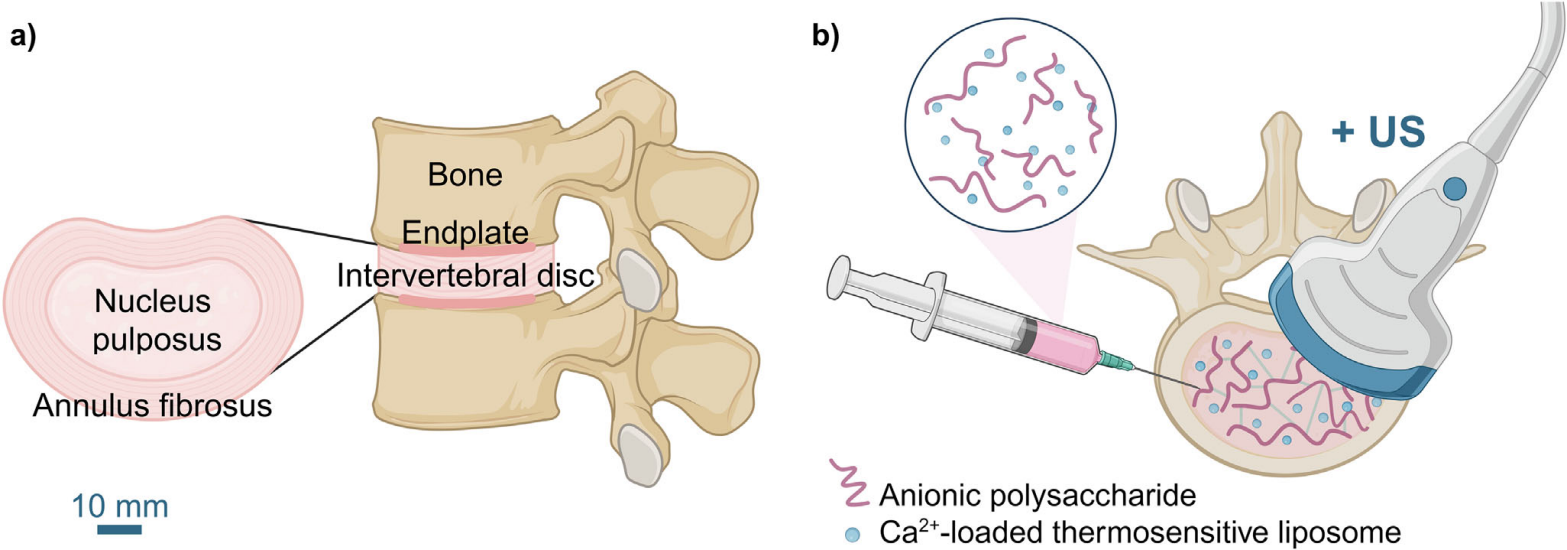
a) Intervertebral disc (IVD) components: annulus fibrosus, nucleus pulposus, and cartilaginous endplates. b) Schematic illustrating the use of an injectable biomaterial for treating IVD degeneration that forms a hydrogel in situ upon ultrasound exposure for treating IVD degeneration. Created in BioRender. Kaesbauer, S. (2025) https://BioRender.com/g04s612 and https://BioRender.com/e66b795.

The IVD is composed of three main tissues (**Figure** **1**a). The nucleus pulposus (NP) is a gelatinous, hydrated matrix of collagen and proteoglycans surrounded by the annulus fibrosus, a connective tissue with primarily type I collagen in the outer annulus and type II collagen in the inner annulus. These are capped by cartilaginous endplates consisting of hyaline cartilage located at the superior and inferior aspects of the vertebral body.^[^ ^8^ ^]^ The structure's complex organization enables it to dissipate complex mechanical loads.^[^ ^9^ ^]^ This function relies on the NP's high water content, the shear resistance of the annulus fibrosus, and the limited swelling capacity of the cartilaginous endplates.^[^ ^9^ ^]^ However, the IVD's biomechanical properties can be substantially impacted by deterioration in the disc's tissues. This process, known as IVD degeneration, is irreversible and involves cellular, biochemical, structural, and mechanical changes including annular tears,^[^ ^10^, ^11^ ^]^ reduced proteoglycan content,^[^ ^12^, ^13^ ^]^ endplate calcification,^[^ ^14^ ^]^ and cell senescence.^[^ ^15^, ^16^ ^]^ IVD degeneration typically begins in the NP, where increased stiffness and reduced permeability make it a key therapeutic target.^[^ ^17^ ^]^

Degeneration typically results in increased production of biomolecules linked to inflammation, a loss in disc height, and pressure on the nervous system, resulting in pain. Conventional treatments, including conservative approaches (physiotherapy, pain medication, rest),^[^ ^18^ ^]^ frequently offer only temporary relief without addressing the underlying disc pathology. Surgical options, such as total disc replacement and spinal fusion,^[^ ^19^ ^]^ are invasive, costly, and often associated with poor long‐term outcomes and complications, such as adjacent segment disease and recurrence of degeneration.^[^ ^20^ ^]^ Emerging regenerative strategies focus on biomaterials and bioengineered scaffolds to support tissue repair and restore disc function.^[^ ^21^, ^22^, ^23^ ^]^ Complementary approaches include cell‐based therapies,^[^ ^21^, ^22^ ^]^ growth factor injections,^[^ ^23^, ^24^ ^]^ exosome, and RNA‐based therapies.^[^ ^25^, ^26^, ^27^, ^28^, ^29^ ^]^ In addition to these emerging strategies, nanotechnology‐based drug delivery systems have shown significant promise for IVD degeneration treatment. These platforms include nanoparticles, microspheres, gene‐nanocomplexes, exosomes, and nanocomposite hydrogels, designed to overcome the avascular and dense extracellular matrix of the IVD by enhancing drug retention, sustained release, and targeted delivery to mitigate inflammation, apoptosis, and matrix degradation.^[^ ^30^, ^31^ ^]^

Despite their potential, nanotechnology‐based drug delivery systems face challenges including cytotoxicity of synthetic materials, complex manufacturing processes, limited gene vector efficiency, and inflammatory degradation byproducts. Exosomes, while biocompatible, suffer from heterogeneity and low yields. Nanocomposite hydrogels require further optimization in mechanical stability and degradation profiles. Moreover, no nanomedicine therapies for IVD disease have yet achieved clinical approval, partly due to inadequate animal models representing human disease progression.

Our ultrasound‐triggered gelation approach integrates polymer solutions with microspheres and calcium‐loaded thermosensitive liposomes to enable minimally invasive, spatiotemporally controlled hydrogel formation within the disc. This strategy localizes therapeutic delivery and scaffold formation, potentially reducing systemic exposure and overcoming challenges of accumulation and repeated administration. Unlike systemic or viral vector‐based nanotechnologies, our system uses well‐established materials in a tunable platform with clear translational potential.

Multiple injectable hydrogel technologies have reached clinical trials. The first attempt was in 1955 when poly(methyl methacrylate) (PMMA) was injected into 14 patients with herniated discs.^[^ ^32^ ^]^ Amongst the injectable biomaterials studied for NP replacement, NuCore (Spine Wave), BioDisc (Cryolife), GelStix (Replication Medical), SaluDisc (SpineMedica), and BIOSTAT BIOLOGX (Spinal Restoration) have received CE, FDA, and/or IDE approval.^[^ ^32^, ^33^ ^]^ Interestingly, NuCore, a protein‐based hydrogel, showed restoration of disc height, which was preserved two years after treatment.^[^ ^33^, ^34^, ^35^ ^]^ However, despite the success of biomaterial design, drawbacks associated with internal migration of the injected material, extrusion, and endplate changes urge the development of new biomaterials for NP replacement.^[^ ^32^ ^]^

Several studies have previously explored hydrogels as biomaterials that could restore the biomechanical properties of NP and IVD function. Both implantable^[^ ^36^, ^37^ ^]^ and injectable^[^ ^34^, ^35^, ^38^, ^39^, ^40^, ^41^ ^]^ systems have been investigated, with the latter being favored as they allow for minimally invasive administration followed by in situ hydrogel formation. Hydrogels comprise either covalent or non‐covalent networks that can be formed through a variety of triggers, with the most common stimuli being UV or blue light irradiation, a temperature change, or self‐assembly following mixing.^[^ ^42^ ^]^ Amongst various covalent crosslinking methods, radical mechanisms are popular as they can be spatiotemporally controlled via light initiation. However, in addition to the delivery of light to deep tissue regions being challenging, radical mechanisms use photoinitiators and generate radicals, both of which can have adverse biological effects. Temperature‐responsive systems rely on hydrogen bonding and hydrophobic interactions holding the network together, which may limit the long‐term in vivo stability required for tissue replacement applications. Notable examples include the use of poly(*N*‐isopropyl acrylamide)‐based thermogels,^[^ ^43^, ^44^, ^45^ ^]^ hyaluronic acid‐based hydrogels,^[^ ^38^, ^41^ ^]^ and photocrosslinkable poly(ethylene glycol) hydrogels.^[^ ^39^ ^]^

To address these challenges, we evaluated the use of ultrasound as an alternative trigger to provide on‐demand gelation for NP regeneration. This approach would benefit from the ability of ultrasound to penetrate deeper tissues relative to UV irradiation, while providing a tunable and adaptable platform with a variety of hydrogelation mechanisms. Several localized heating techniques have been explored for triggering gelation or drug release, including magnetic hyperthermia, radiofrequency (RF) heating, and near‐infrared (NIR) light irradiation, each with distinct advantages and limitations. Magnetic hyperthermia employs alternating magnetic fields to heat magnetic nanoparticles but faces challenges such as limited penetration depth, off‐target heating, and concerns over nanoparticle retention and biocompatibility, restricting its suitability for deep tissues like the IVD.^[^ ^46^, ^47^, ^48^ ^]^ RF heating offers deeper tissue penetration and is used clinically in tumor ablation; however, its precision is limited by tissue electrical heterogeneity, complicating targeting and material design.^[^ ^49^, ^50^ ^]^ NIR light, often combined with photothermal agents, enables minimally invasive heating but suffers from shallow tissue penetration and attenuation by overlying tissues, limiting its use for deep anatomical sites such as the lumbar spine.^[^ ^51^ ^]^ In contrast, focused ultrasound provides non‐invasive, precise energy delivery with millimeter‐scale focal accuracy to deep tissues, overcoming the penetration constraints of other modalities.^[^ ^52^ ^]^ Focused ultrasound can be integrated with real‐time imaging modalities like MRI or ultrasound for accurate targeting and temperature monitoring, enhancing safety and treatment efficacy.^[^ ^53^, ^54^ ^]^ Furthermore, the dynamic modulation of focused ultrasound parameters enables the creation of tailored gelation patterns necessary for complex structures, such as the IVD. These unique capabilities position ultrasound‐triggered gelation as a promising strategy for minimally invasive, localized treatment of deep, load‐bearing tissues. In addition, the use of ultrasound provides precise control over the gelation kinetics in comparison to shear‐thinning and self‐healing injectable hydrogels.^[^ ^55^, ^56^ ^]^

Studies using ultrasound as a source for inducing the formation of a 3D network have relied on the use of hazardous radical initiators,^[^ ^57^ ^]^ organic solvents,^[^ ^58^ ^]^ or extremely high ultrasonic pressures,^[^ ^57^ ^]^ all of which may limit their clinical applicability. A promising alternative lies in the use of thermally responsive liposomes, which are lipid‐based vesicles that allow the encapsulation of molecules in their aqueous core and subsequent on‐demand release by heating to the target temperature.^[^ ^59^, ^60^, ^61^, ^62^, ^63^, ^64^ ^]^ The lipid composition can be tuned to establish a transition temperature for cargo release under mild hyperthermia induced by low‐power ultrasound, which has been safely demonstrated both preclinically and clinically.^[^ ^62^, ^63^, ^64^ ^]^ While thermosensitive liposomes have been widely used for ultrasound‐triggered drug release, especially in oncology, their application for in situ gelation in deep, load‐bearing tissues remains limited. Prior studies demonstrated ultrasound‐triggered enzymatic gelation and used focused ultrasound to construct soft tissue biomaterials via crosslinker release from thermosensitive liposomes.^[^ ^65^, ^66^ ^]^ However, these approaches were not designed for mechanically demanding environments like the spine, used ultrasound frequencies in the kHz or high MHz range, and relied on different gelation mechanisms (e.g., enzymatic‐based).

Our system uses 0.95–1.25 MHz focused ultrasound to trigger calcium release from thermosensitive liposomes, initiating alginate gelation directly within the NP. This enables spatially controlled gel formation at clinically relevant depths, offering both mechanical reinforcement and minimally invasive delivery. Clinical precedents for ultrasound‐triggered liposome activation in internal organs, such as the TARDOX trial for treatment of liver tumors, support the translational potential of our approach.^[^ ^62^, ^67^ ^]^

Here, we present a novel ultrasound‐triggered gelation system for IVD repair through augmentation (Figure 1b), addressing key challenges in biomaterial formulation, ultrasound activation, and functional validation. Our approach involves a two‐part injectable hydrogel precursor system combining calcium‐loaded thermosensitive liposomes with a sodium alginate polymer solution.^[^ ^65^ ^]^ The liposomes are designed to release the crosslinking agent, i.e., calcium ions, upon ultrasound activation to enable spatiotemporally controlled crosslinking of alginate for in situ clinician‐controlled implant formation.

The following sections describe the optimization of the biomaterial formulation and ultrasound exposure parameters, as well as the development of thermal and acoustic methods for real‐time gelation process control. We present results from in vitro ultrasound‐triggered gelation, cytocompatibility, and mechanical testing, followed by ex vivo proof‐of‐concept testing in bovine discs under simulated physiological loading. This study demonstrates the feasibility of needle injection, ultrasound activation, and biomechanical assessment in a clinically relevant model.

## 2 Results and Discussion

### 2.1 Biomaterial Characterization

We optimized a hydrogel precursor system: a free‐flowing aqueous solution consisting of thermally sensitive liposomes and an anionic polysaccharide, namely sodium alginate. The liposomes consist of 99:1 molar ratio of 1,2‐dipalmitoyl‐sn‐glycero‐3‐phosphocholine (DPPC) and 1,2‐distearoyl‐sn‐glycero‐3‐phosphoethanolamine‐*N*‐{methoxy[poly(ethylene glycol)]‐2000} (ammonium salt) (DSPE‐PEG2000). This system was designed to respond to mild hyperthermia by forming a physically crosslinked calcium alginate hydrogel.

**Figure 2:**
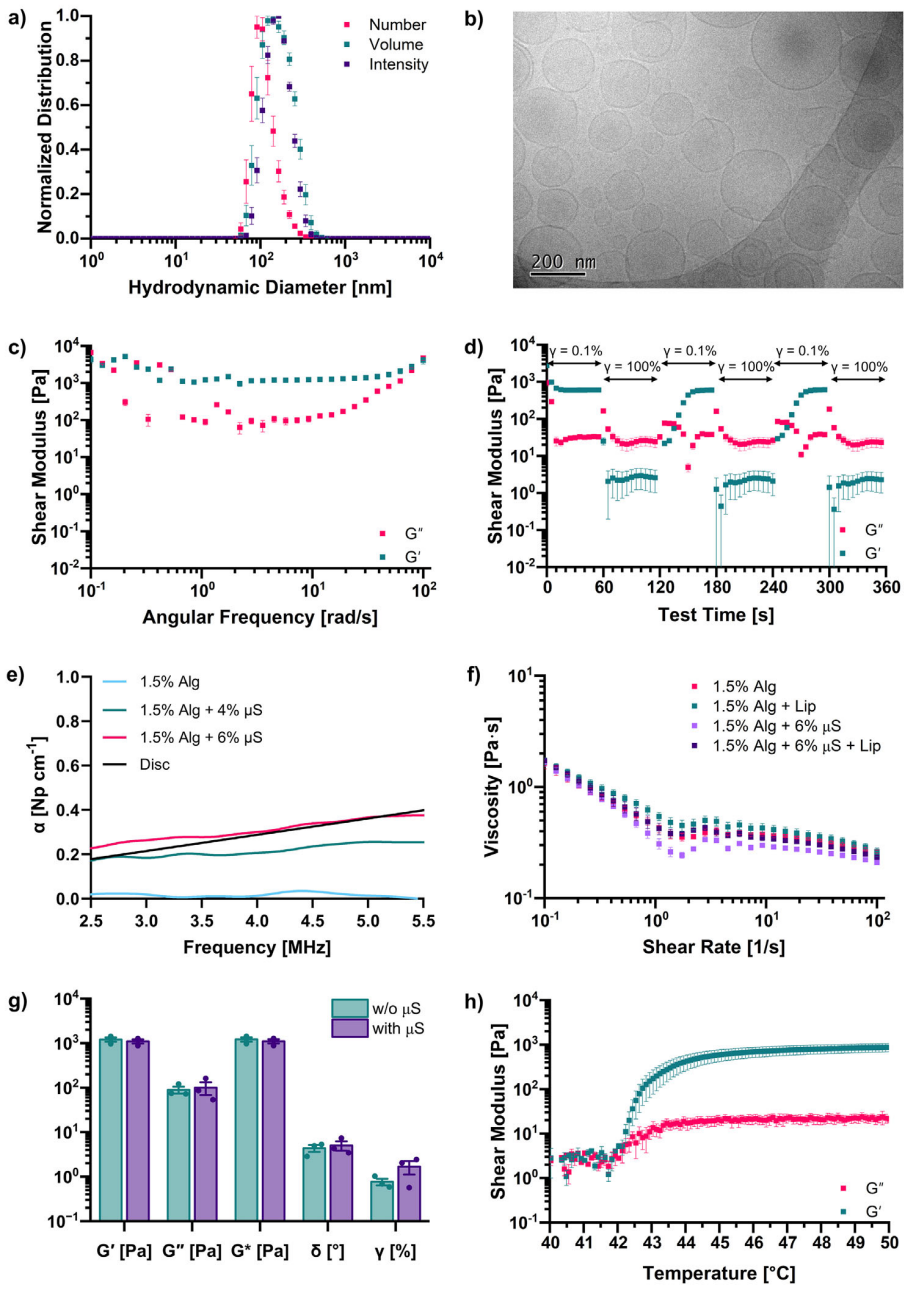
a) Normalized number, intensity, and volume distributions for the liposome formulation produced via the interdigitation‐fusion method at a concentration of 0.5 mg mL^−1^ in iso‐osmotic buffer (mean ± S.E. based on *n* = 3 liposome batches). b) CryoTEM image of the liposome formulation at 20 mg mL^−1^ in iso‐osmotic buffer at 20 °C (*n* = 1 liposome batches). c) Frequency‐sweep measurements on the hydrogels made of Alg_1_ at 37 °C and alginate concentration of 1.5% wt/v (mean ± S.E. based on *n* = 3 liposome batches). d) Strain recovery measurements on the hydrogels made of Alg_1_ at 37 °C and alginate concentration of 1.5% wt/v (mean ± S.E. based on *n* = 3 liposome batches). e) Attenuation estimates with purified microspheres (µS). Samples consisted of 1.5% wt/v Alg_1_ and two concentrations of purified µS: 4% wt/v and 6% wt/v, as well as a sample consisting of just 1.5% wt/v Alg_1_ without any µS. The data plotted is the mean average of three individual measurements. The power law fit for ex vivo bovine IVD material is shown in black. f) Flow‐sweep measurements at 25 °C on the hydrogel precursor consisting of Alg_1_ at 1.5% wt/v with and without µS (mean ± S.E. based on *n* = 3 liposome batches). g) Properties resulting from rheological measurements on the hydrogels made of Alg_1_ at 37 °C and alginate concentration of 1.5% wt/v with and without µS (mean ± S.E. based on *n* = 3 liposome batches). h) Temperature ramp measurements on the hydrogel precursor consisting of Alg_1_ and µS (1.5% wt/v and 6%, respectively), indicating gelation upon heating (mean ± S.E. based on *n* = 3 liposome batches).

The purified liposomes produced via the interdigitation‐fusion method were characterized using dynamic light scattering (DLS) to determine their hydrodynamic diameter, with normalized distributions shown in **Figure** **2**a. The DLS results indicate narrow size distributions with a *Z*‐average size and polydispersity index of 152 ± 2 (mean ± S.E.) nm and 0.10 ± 0.01 (mean ± S.E.), respectively. The DLS size distributions of liposomes produced with different approaches (see Section 4) can be found in Figure S1a–c (Supporting Information), indicating good agreement between the different methods employed. The encapsulated calcium concentration was determined to be between 10–15 mm, depending on the liposome production method, with the highest values achieved via the interdigitation‐fusion method, as expected and shown in Figure S1d (Supporting Information). In addition, we investigated the thermally induced calcium release from the liposomes, and we observed that the percentage of released calcium increased from 6.1 ± 0.3 to 72.3 ± 1.3% as the temperature was raised from normal body temperature (37 °C) to mild hyperthermia (39 °C), as shown in Figure S2 (Supporting Information). Up to 85% of the total encapsulated calcium was released at 40 °C, with no further release being observed upon further heating to 45 °C. This liposome formulation has previously been shown to be stable against aggregation and passive calcium release at 25 °C for 5 days,^[^ ^65^ ^]^ while stability under storage conditions for one month was confirmed (Figure S3, Supporting Information).

Liposome samples at 20 and 42 °C were analyzed via cryogenic transmission electron microscopy (cryo‐TEM), which confirmed the presence of unilamellar vesicles (Figure 2b; Figures S4 and S5, Supporting Information). Analysis of particle size revealed a mean diameter of 165 nm and a standard deviation of 74 nm in one sample. Upon heating to 42 °C, which is above the melting temperature of the DPPC lipids, the mean particle size reduced to 136 with a standard deviation of 70 nm, and the number of faceted particles increased, a trend previously observed and reported for thermosensitive liposomes.^[^ ^68^ ^]^

#### 2.1.1 Base Biomaterial Characterization

To demonstrate hydrogelation using calcium‐loaded thermosensitive liposomes, we focused on investigating anionic polysaccharides, which are known to form ionically crosslinked networks in the presence of divalent cations.^[^ ^65^, ^69^, ^70^ ^]^ Alginate is the most well‐known member of this family, but poly(galacturonic acid) (PGA) and pectin have also been reported to form hydrogels in the presence of divalent cations^[^ ^69^, ^71^, ^72^, ^73^, ^74^, ^75^, ^76^, ^77^ ^]^; the structure of the polymers is shown in Figure S6 (Supporting Information).

The gelation of these anionic polysaccharides can be described by the “egg‐box” model, according to which two carboxylic groups originating from different polymer chains. coordinate to the same divalent cation.^[^ ^69^ ^]^ Therefore, we investigated a library of anionic polysaccharides that, when mixed with calcium‐loaded thermosensitive liposomes, could potentially undergo gelation through thermally induced cargo release. The gelation was inspected visually via the tube‐inversion method, while the shear mechanics of the formed hydrogels were investigated using rheology (Figure S7, Supporting Information). We observed that PGA produced hydrogels with the highest shear storage modulus. However, the polymer precursor solutions faced issues of precipitation and poor long‐term stability. Three different alginates from different sources and of different viscosities, as detailed in the Materials section, were mixed with liposomes containing 15 mm calcium chloride, and upon heating, hydrogels were formed. It was observed that Alg_1_ hydrogels exhibited a higher shear storage modulus than those produced with Alg_2_ and Alg_3_, indicating that slight changes in the polymer structure (Figure S8, Supporting Information) play a role in the rheological properties of the gels. Pectin solutions did not undergo gelation under the conditions tested.

We therefore chose to use Alg_1_ precursor, and following optimization of the alginate concentration (Figure S7d, Supporting Information), we established an optimal formulation of 1.5% w/v sodium alginate with 15 mM calcium‐loaded liposomes in 0.3 m NaCl. The formulation at 20 °C was imaged via cryoTEM, which confirms that the new conditions, e.g., mixing with the aqueous sodium alginate solution, do not affect the liposome structure (Figure S9, Supporting Information). Samples were cast in cylinders and subjected to in‐depth rheological characterization. First, a strain sweep was used to identify the linear viscoelastic region (LVR) (Figure S10a, Supporting Information), with a value of 0.1% strain chosen for a frequency sweep (Figure 2c). This study confirmed the presence of a hydrogel with the shear storage modulus (Gˊ) exceeding the shear loss modulus (G˝). The gel stiffness was relatively independent of the frequency applied, with the elastic modulus remaining at values ∼1 kPa across the angular frequency range tested. Strain‐recovery measurements were performed on the hydrogels by alternating the applied strain from 0.1% (within the LVR) to either 100% or 10%, which are both outside the LVR, where the sample flows (G˝>Gˊ) (Figure 2d; Figure S10b, Supporting Information), respectively). These results show the recovery of the biomaterial, as the initial moduli values are reached upon switching from high strain to low strain.

#### 2.1.2 Acoustic Attenuation Enhancement with Microspheres

To safely control the in situ heating process, the ultrasound absorption of the implanted biomaterial should match or exceed that of native tissue. Initial tests showed relatively low absorption in the precursor hydrogel. This was addressed by adding purified glass microspheres (µS) to the hydrogel, as inhomogeneities in the medium can enhance acoustic energy absorption through viscous or thermal processes.^[^ ^78^, ^79^ ^]^ Adding 4 or 6% w/v glass microspheres to 1.5% w/v Alg_1_ increased the attenuation (used as an approximate measurement of absorption) to physiological levels (Figure 2e).

Based on the collective data presented in this section, the formulation consisting of 1.5% wt/v Alg_1_ and 6% wt/v microspheres was chosen for further testing. The ultrasonic attenuation fit for this formulation was:

(1) $$\alpha_{hydrogel}=0.13+0.04f$$

whereas the fit for the healthy bovine disc was:

(2) $$\alpha_{bovine}=0.07f^{1.02}$$

where *f* is the frequency (MHz) and attenuation is in units of Np cm^−1^. The chosen formulation exhibited a 2.25‐fold higher attenuation at 1.1 MHz (the approximate frequency of ultrasound used in the treatments – see Section 2.2) than non‐degenerated bovine disc material and should therefore enable preferential heat deposition in the injected material, lower required incident field intensities, and reduced risk of off‐target hyperthermia.

#### 2.1.3 Characterization of Biomaterial with Microspheres

Following attenuation optimization, rheology was used to characterize the precursor solution and the resulting hydrogel with and without microspheres. As injectability is an important feature for minimally invasive biomedical applications, we investigated the injectability properties of the precursor solutions with and without liposomes and/or µS. The trends shown in Figure 2f confirm their shear‐thinning properties (decrease in viscosity at increased shear rates), and thus the injectability of our biomaterial, indicating that the shear‐thinning properties are preserved when incorporating microspheres. We also compared the shear moduli and the phase angle determined by frequency sweeps, as well as the strain at the limit of the LVR determined by amplitude sweeps (Figure 2g). No difference in the shear moduli or phase angles was observed, indicating that the addition of microspheres does not impact the stiffness of the hydrogels within the LVR. Interestingly, the LVR was extended to higher values with the addition of the microspheres. Hydrogel precursor samples containing microspheres were also subjected to temperature ramps (Figure 2h). Gˊ and G˝ are low at body temperature but increase in magnitude close to the melting temperature of the DPPC lipids, with a crossover observed at ≈42.2 °C, in agreement with the visual observations. The control sample, i.e., 1.5% wt/v Alg_1_ solution containing 6% w/v microspheres without liposomes, showed no gelation under the same test conditions (Figure S10c, Supporting Information).

#### 2.1.4 Evaluation of In Vitro Biocompatibility

**Figure 3:**
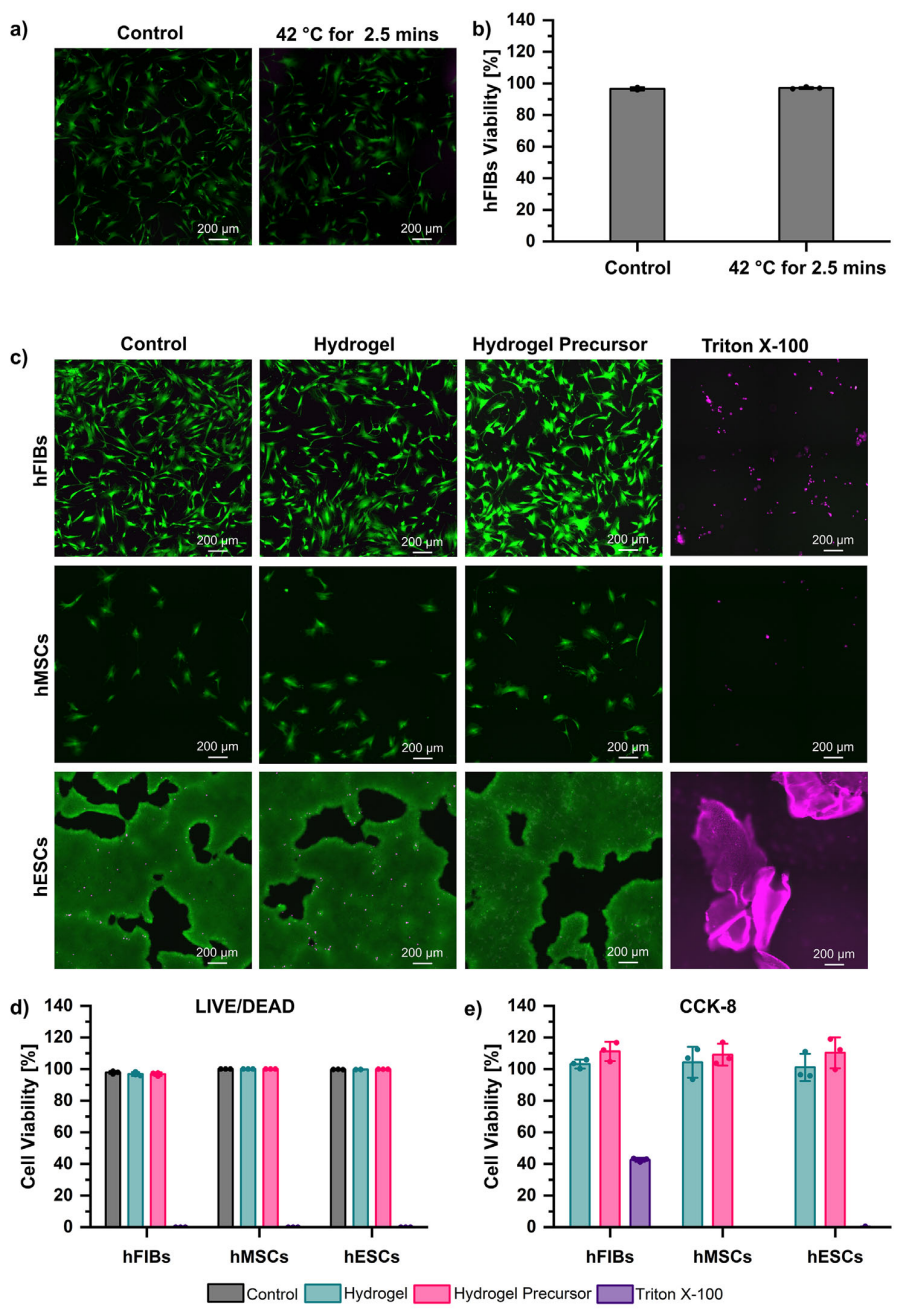
a) LIVE/DEAD staining images of human fibroblasts (hFIBs) heated to 42 °C for 2.5 min (right) against the control (no heating, left). b) Viability (%) of hFIBs after heating to 42 °C for 2.5 min (right) against the control (no heating, left), as determined by LIVE/DEAD staining. c) LIVE/DEAD staining images of hFIBs, human mesenchymal stem cells (hMSCs) and human embryonic stem cells (hESCs) exposed to the hydrogel (1.5% wt/v Alg_1_ seeded with calcium‐loaded liposomes (10–15 mm calcium chloride) and 6% µS) and hydrogel precursor (1.5% wt/v Alg_1_ seeded with calcium‐loaded liposomes (10–15 mm calcium chloride) and 6% µS). Negative and positive controls correspond to untreated cells and cells treated with Triton X‐100, respectively. d) Quantified viability (%) of hFIBs, hMSCs, and hESCs after exposure to the hydrogel, hydrogel precursor, or Triton X‐100 (death control) and control (untreated cells, no exposure), for which representative LIVE/DEAD images are shown in (c). e) Viability (%) relative to the control (untreated cells, no exposure) of hFIBs, hMSCs, and hESCs after exposure to the hydrogel (1.5% wt/v Alg_1_ seeded with calcium‐loaded liposomes (10–15 mm calcium chloride) and 6%µS), hydrogel precursor (1.5% wt/v Alg_1_ seeded with calcium‐loaded liposomes (10–15 mm calcium chloride) and 6%µS), and Triton X‐100 (all normalized to control), as determined by CCK‐8 metabolic assay.

Cell viability was investigated to confirm the safety and biocompatibility of both the heat‐based activation method and the biomaterial. The method involves transient heating of the material to 42 °C for 2.5 min to trigger calcium release and induce hydrogelation. To evaluate the potential effects of this heating step, primary human fibroblasts (hFIBs) were exposed to the same thermal conditions (42 °C for 2.5 min) and compared against a no‐heat control. Cell viability was then assessed via LIVE/DEAD staining (**Figure** **3**a,b), which indicated preserved survival of the cells and their compatibility with the proposed methodology.

In addition, the in vitro biocompatibility of the biomaterial was investigated using multiple cell lines with varying sensitivities. Three representative cell lines were selected in order of increasing sensitivity to their environment: i) hFIBs, found in connective tissue (relatively resilient), ii) human mesenchymal stem cells (hMSCs), found in circulation (moderately sensitive), and iii) human embryonic stem cells (hESCs, highly sensitive). All cell lines were exposed for 24 h to either the hydrogel or its precursor (1.5% wt/v Alg_1_ solution seeded with calcium‐loaded liposomes (10–15 mm calcium chloride) containing 6% w/v microspheres). Cell viability was quantified using LIVE/DEAD staining (Figure 3c,d) and a CCK‐8 metabolic assay (Figure 3e). Triton X‐100‐treated cells and untreated cells were employed as positive and negative controls, respectively. LIVE/DEAD staining showed preserved morphology and viability in cells exposed to the biomaterial, whereas Triton X‐100 induced extensive cell death. These results were consistent with metabolic assay data, which revealed no meaningful differences between the biomaterial and control groups across all three cell lines. The inclusion of hESCs, among the most sensitive human cell types to environmental changes, provides a stringent assessment of biocompatibility, further confirming that the biomaterial formulation does not adversely affect cell survival. These results demonstrate that the proposed methodology and biomaterial do not cause cytotoxicity in a representative range of human cells, supporting their potential for future translation of this biotechnology.

### 2.2 Ultrasound Parameter Optimization for In Vitro Heating

#### 2.2.1 Therapeutic Source Frequency

To evaluate the effects of source frequency on heating rate and consistency, three ultrasound frequencies (0.95, 1.1, and 1.25 MHz) were chosen based on the radial response profile from the transducer calibration. Experimental ultrasound parameters included a fixed peak negative pressure (PNP) of 1.6 MPa, a 2 ms burst period, and a varying number of cycles (1650, 1911, 2171 for 0.95, 1.1, 1.25 MHz, respectively) to maintain a consistent duty cycle (i.e., the percentage of time ultrasound is generated within each burst period) of ≈87%. This high duty cycle provided substantial time‐averaged ultrasound energy deposition to maximize the heating rate. Frequency assessments were conducted on a single uncrosslinked hydrogel formulation: 1.5% wt/v Alg_1_ in 0.3 m NaCl, containing 6% wt/v purified microspheres (no liposomes).

**Figure 4:**
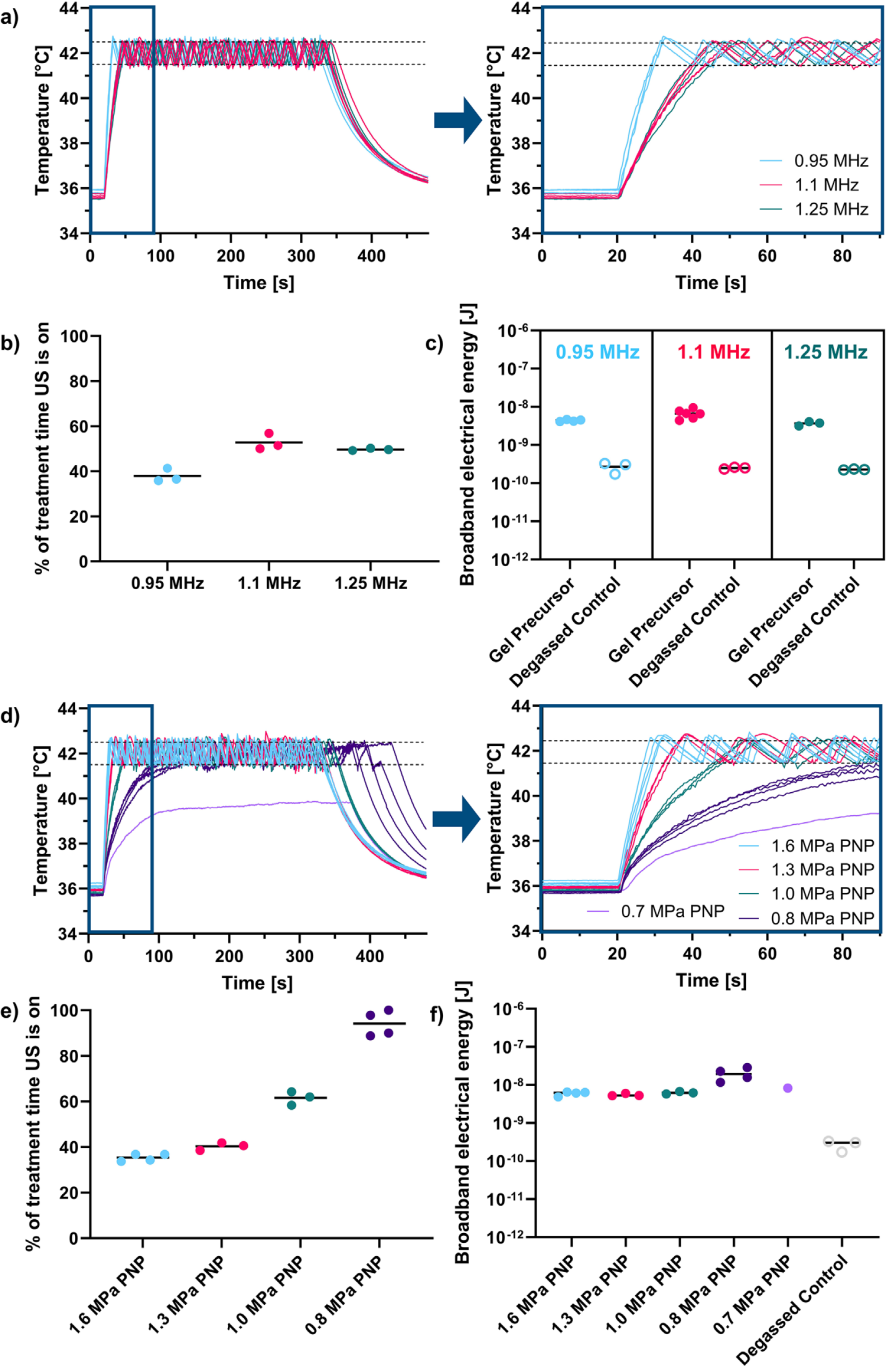
a) Heating curves for samples with 1.5% wt/v Alg_1_ in 0.3 m NaCl and 6% wt/v microspheres at frequencies of 0.95, 1.1, and 1.25 MHz. The right panel zooms in on the first 90 s. b) Proportion of treatment time with ultrasound active at each frequency. c) Broadband PCD signal energies at each frequency, calculated until the temperature first reaches the upper threshold. d) Heating curves for samples with 1.5% wt/v Alg_1_ in 0.3 m NaCl and 6% wt/v microspheres, varying with pressure at 0.95 MHz; the right panel zooms in on the first 90 s. e) Proportion of ultrasound on‐time for each pressure condition. f) Broadband PCD signal energies for each pressure, calculated until the temperature first reaches the upper threshold. Curves and points represent individual repeats.

**Figure** **4**a shows temperature variations over time using an automated temperature‐feedback algorithm for controlling the ultrasound exposure. Dashed lines represent the target temperature range, which the algorithm maintained effectively at constant pressure. We observed an inverse relationship between heating rate and frequency, suggesting a dominant effect of increased beam volume (inversely related to frequency‐cubed) at low frequencies. Variability in heating curves is likely due to thermocouple placement sensitivity, especially as the focal spot size decreases at higher frequencies. Simulations indicated that at 1.1 MHz, the temperature could drop by 0.8 and 2.2 °C for radial errors of 0.5 and 1.0 mm, respectively, increasing to 1.1 and 2.6 °C at 1.25 MHz. Incidental heating of the sample holder may also contribute.

We evaluated ultrasound exposure within a 5‐min treatment window with the aim of facilitating a more efficient treatment protocol. For non‐degassed liposome‐free samples, Figure 4b shows that ultrasound‐on time increased with frequency, from (28 ± 4)% at 0.95 MHz to (48 ± 2)% at 1.25 MHz, underscoring the heating efficiency of the lowest frequency. Using passive cavitation detection (PCD), broadband cavitation activity, suggesting inertial cavitation, was measured for all samples and compared to a degassed water control sample (i.e., a control with no cavitation) (Figure 4c). Broadband energies for the initial ultrasound exposure (until the temperature first reached 42.5 °C) were similar across frequencies despite varying times to reach the temperature threshold. The comparable broadband energy needed for a 42.5 °C temperature rise, alongside consistent cavitation energies, on‐times, and heating rates, suggests that these trials were conducted within a reliable cavitation‐enhanced heating parameter range.^[^ ^80^ ^]^

#### 2.2.2 Therapeutic Source Pressure

A pressure sweep was conducted to evaluate the effect of pressure on heating rate and consistency, using 0.95 MHz, a 2 ms burst period, 1650 cycles (≈87% duty cycle), and pressures from 0.7 to 1.6 MPa PNP. The highest pressure was selected from pilot results showing effective controlled heating, while lower pressures were tested to identify the minimum safe and reproducible heating threshold. In the absence of nonlinearity, the ultrasound‐mediated heat deposition rate (q) is quadratically dependent on the pressure^[^ ^81^ ^]^:

(3) q=2aI

where *a* is the local absorption, and for a progressive plane wave^[^ ^81^ ^]^:

(4) $$I=p_{rms}^{2}/Z$$

where *Z* is the characteristic impedance of the material and *p_rms_* is the root mean square pressure.

The effect of pressure on sample heating was evaluated at 1.6, 1.3, 1.0, 0.8, and 0.7 MPa PNP. Only one sample was collected at 0.7 MPa, as this condition did not provide sufficient energy to reach the upper temperature threshold of 42.5 °C, deeming it unsuitable for this application. Figure 4d shows that the heating rate decreased with lower pressure, as expected, with 0.8 MPa as the minimum required to reach the target temperature. Greater variability in heating rate and cavitation energy profiles was observed at 0.8 MPa (Figure 4d,f), as heat dissipation becomes more significant at lower heating rates.

Though the time to target temperature increased as pressure decreased (Figure 4e), broadband cavitation energy remained consistent across the highest three pressures (Figure 4f). For pressures below the threshold, heating energy continued to accumulate without reaching the upper limit. Ultrasound on‐time approached 100% at 0.8 MPa, increasing from (35 ± 2)% at 1.6 MPa to (94 ± 6)% at 0.8 MPa (Figure 4e). This supports using higher pressures with lower frequencies for stable, safe ultrasound‐triggered gelation.

Cumulatively, these frequency and pressure sweeps identified the optimal exposure parameters for the precursor hydrogel (1.5% wt/v Alg_1_ in 0.3 m NaCl with 6% wt/v microspheres) as 0.95 MHz, 1.6 MPa PNP, 1650 cycles, and a 2 ms burst period (87% duty cycle).

### 2.3 Thermometry‐Guided In Vitro Gelation

#### 2.3.1 Temperature and Cavitation Profiles

**Figure 5:**
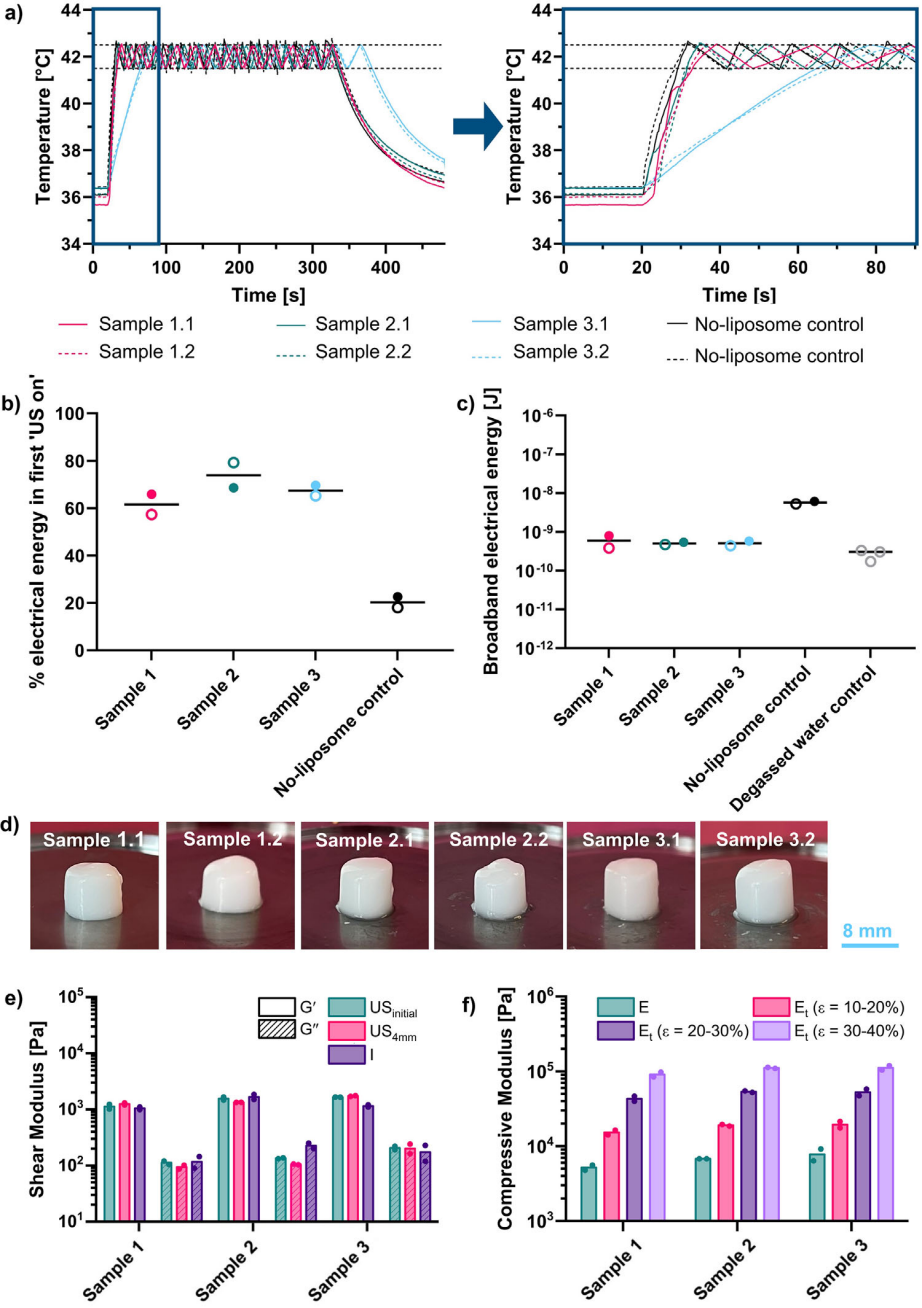
Samples of 1.5% wt/v Alg_1_ in ≈0.3 m NaCl, 6% wt/v microspheres, and thermosensitive liposomes containing 10–15 mm calcium chloride. Three liposome batches were used, and samples are labeled as x.y, where x corresponds to the liposome batch, and y corresponds to the replicates within the same liposome batch. a) Heating curves for samples and no‐liposome controls (right panel highlights the first 90 s). b) Proportion of total PCD signal energy emitted during the initial ultrasound‐on phase. c) PCD signal broadband energy for hydrogel samples, no‐liposome controls, and degassed water controls (baseline) calculated until the upper temperature threshold is first reached. Curves and data points represent individual repeats. d) Photographs of the hydrogels produced upon temperature‐guided ultrasound exposure (scale bar = 8 mm). e) Shear moduli of the three different samples as demonstrated by frequency sweep measurements. The samples produced by ultrasound exposure were tested as extracted (US_initial_) and after processing to 4 mm height (US_4mm_), and they were compared to the hydrogels prepared in an incubator (I_1mm_) (n = 2 for different samples with the same liposome batch). f) Compressive moduli, i.e., Young's moduli (E) and tangent moduli (E_t_), as demonstrated by unconfined compression tests on the samples produced by ultrasound exposure (*n* = 2 for different samples with the same liposome batch).

In vitro ultrasound‐triggered gelation was next demonstrated using the optimized parameters (0.95 MHz, 1.6 MPa PNP, 1650 cycles, and a 2 ms burst period, constituting an 87% duty cycle) applied for 5 min to the optimized biomaterial (1.5% wt/v Alg_1_, 15 mm calcium‐loaded liposomes, and 6% wt/v microspheres). Ultrasound‐triggered gelation was tested in duplicate for three different batches of liposomes, thus, the samples are labeled as x.y, where x corresponds to the liposome batch, and y corresponds to the replicates within the same liposome batch. Heating curves showed that two batches (sample 1 and sample 2) were similar to duplicates of a no‐liposome control but with slightly longer rise times (i.e., the time to reach the upper temperature threshold of 42.5 °C) while the third batch (sample 3) showed delayed heating, possibly due to sub‐optimal liposome fabrication and thus Ca^2+^ release, or microsphere sedimentation (**Figure** **5**a).

Cavitation signal analyses revealed that 60–80% of the total energy was emitted in the first ultrasound exposure period for liposome‐containing samples, while controls emitted only 20% in this period (Figure 5b). The cavitation signal energy associated with reaching 42.5 °C was again comparable across hydrogel samples, but no‐liposome controls showed ≈10 times higher initial broadband energy than liposome‐containing samples (Figure 5c). Despite delayed heating for sample 3 (Figure 5a), gelation occurred reliably in all samples, as shown in Figure 5d.

#### 2.3.2 Rheology and Unconfined Compression Tests

During the proof‐of‐concept study, three different liposome batches were used, and two gels were produced from each batch to investigate the reproducibility of the experiment as detailed in the previous section. The photographs in Figure 5d show the produced hydrogels made from 1.5% wt/v Alg_1_ in ≈0.3 m NaCl, 6% wt/v microspheres, 10–15 mm calcium chloride, as extracted from the ultrasound exposure sample holders. As the hydrogels maintained their cylindrical shape as a result of the geometry of the sample holders, these results indicate that ultrasound has been successfully used to trigger gelation. The intact hydrogel samples were subjected to frequency and amplitude sweeps (representative graphs for Sample 1 in Figure S11, Supporting Information). Similar observations to the incubator‐treated samples discussed in the previous section (Figure 2c; Figure S10a, Supporting Information) were made. Specifically, the samples show viscoelastic behavior when subjected to frequency and amplitude sweep measurements, with the Gˊ exceeding G˝ at low frequencies and strains, indicating the presence of a hydrogel. At higher frequency and/or strain values, a crossover is observed (G˝ > Gˊ), and the samples behave as a liquid. However, the side of the gel closest to the therapeutic source (the top side in each photograph) showed incomplete gelation, potentially because of the acoustic radiation force distributing material in the direction of sound propagation or excess water within the sample holder due to pre‐wetting the sample holder to minimize gas bubbles within the biomaterial. Therefore, to obtain better contact with the top plate of the rheometer and thereby improve the reliability of the results, the gels were cut to a height of 4 mm to provide a flat top surface for contact with the top parallel plate on the rheometer (Figure S12a, Supporting Information). The cut samples were subsequently tested by rheology, and the trends were similar to those observed for uncut Sample 1 in Figure S11 (Supporting Information), with hydrogel formation confirmed at low amplitude and frequency values. As can be seen in Figure 5e, comparable values of shear storage (Gˊ) and shear loss (G˝) moduli are observed for the ultrasound‐treated samples (both intact and cut), and the incubator‐treated samples, with values ≈1 kPa.

Lastly, unconfined compression tests were performed on the hydrogel samples, and the results are summarized in Figure 5f and Figure S12b (Supporting Information). The measured Young's modulus (E) of 5.2–8.0 kPa was lower than reported for ex vivo human NP material in the literature (64.9 ± 4.1 kPa).^[^ ^82^ ^]^ It is worth noting that the values of maximum compressive strain before failure are much higher than the target minimum of 25% to 30% compression strain, which is comparable to the physiological strain experienced by the NP. As seen in Figure S12a (Supporting Information), the slicing revealed a void in two out of the six hydrogels, most likely due to the formation of a gas bubble at or near the thermocouple tip during the ultrasound treatment. Photographs of the hydrogels after unconfined compression tests revealed that the voids may have acted as initiation points for failure (Figure S12c, Supporting Information). This motivated the pursuit of a gelation monitoring technique that avoided invasive thermometry.

### 2.4 Cavitation‐Guided Automated Ultrasound Protocol

#### 2.4.1 Rationale

**Figure 6:**
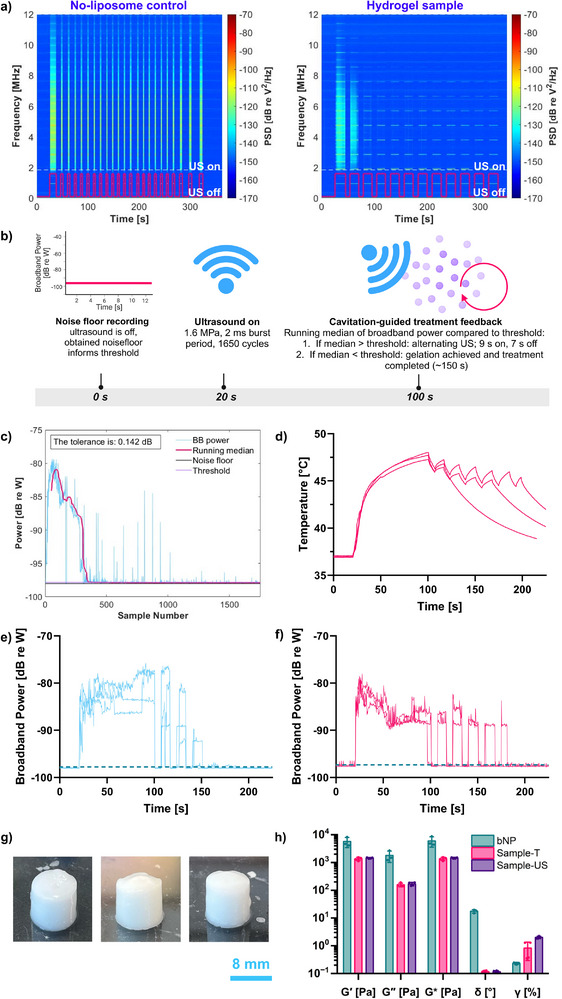
a) Cavitation signal spectra over a ≈5‐min exposure show the reduction of broadband activity (vertical lines) in liposome‐containing hydrogel samples compared to no‐liposome controls. b) Timeline of the cavitation‐guided ultrasound protocol: a 20‐s baseline was followed by 80 s of ultrasound, after which real‐time cavitation monitoring began. Ultrasound continued in a 9‐s on/7‐s off cycle until the broadband median emissions dropped below a set threshold, indicating gelation completion. Created in BioRender. Kaesbauer, S. (2025) https://BioRender.com/i15d538. c) Logic for cavitation‐guided gelation: broadband noise was isolated by removing harmonics and ultraharmonics from the total signal. A running median was compared against a threshold based on baseline noise plus tolerance. d) Temperature curves for liposome samples using the cavitation‐guided protocol, with temperature monitoring to ensure safe heating (*n* = 3 samples). e) Cavitation data (*n* = 3 samples with the same liposome batch) for liposome‐containing samples without a thermocouple; after *t* = 100 s, ultrasound was cycled on/off as long as broadband power exceeded the threshold (blue dotted line). f) Cavitation data corresponding to temperature curves in (d), after *t* = 100 s, ultrasound was cycled on/off as long as broadband power exceeded the threshold (blue dotted line). g) Photographs of the hydrogels produced upon using the cavitation‐guided ultrasound gelation protocol, corresponding to the cavitation data in (e) (scale bar = 8mm). h) Comparison between the rheological properties of the bovine nucleus pulposus (bNP, mean ± S.E. based on *n* = 3 bovine discs), the ultrasonically triggered hydrogels (Sample‐US, mean ± S.E. based on *n* = 4 samples with the same liposome batch), and the hydrogels prepared in an incubator (Sample‐T, mean ± S.E. based on *n* = 4 samples with the same liposome batch).

In optimizing the ultrasound treatment and assessing in vitro gelation, we observed that the broadband cavitation signal energy in liposome‐containing hydrogels was comparable during the first ultrasound phase (until reaching 42.5 °C), regardless of rise time. Notably, the energy levels in the control (no‐liposome) samples were ≈10 times higher. PCD signal spectrograms, shown in **Figure** **6**a, revealed distinct spectral patterns: harmonic emissions (horizontal lines) and broadband emissions (vertical lines) in response to the ultrasound signal. In liposome‐containing samples, broadband emissions ceased within ≈80–130 s, while in no‐liposome controls, cavitation remained steady. This finding was likely due to rapid gelation‐induced biomaterial stiffening in the liposome samples, which acted to reduce cavitation.^[^ ^83^ ^]^ This rapid reduction in cavitation energy suggested that the treatment time could be reduced while still achieving efficient gelation. Additionally, to avoid thermocouple‐induced artifacts, we explored a new treatment guidance protocol based on cavitation rather than thermometry.

#### 2.4.2 Protocol Adjustments

Custom MATLAB code was updated to monitor broadband cavitation emissions in real‐time as indicators of gelation progress. The protocol (Figure 6b) consisted of a 20‐s baseline recording, followed by ultrasound activation for 80 s, after which cavitation monitoring began. A cavitation threshold was set based on baseline noise plus a tolerance factor (Figure 6c). The running median of broadband emissions over the last 50 samples was continuously compared to this threshold. If emissions exceeded the threshold, the ultrasound continued in a 9‐s on/7‐s off cycle. When emissions fell below the threshold, indicating gelation, the treatment was concluded.

#### 2.4.3 Experimental Confirmation

Results in Figure 6d–g confirm the effectiveness of this cavitation‐guided protocol. Initial temperature readings showed a ≈10 °C rise, stabilizing around 45 °C (Figure 6d). Additional trials without the thermocouple (Figure 6e) showed similar cavitation patterns to those for which temperature data was collected (Figure 6f). The on/off ultrasound cycles continued until broadband emissions dropped below the threshold, with treatment ceasing within 80–150 s, confirming consistent treatment without overheating.

Thermal safety of the exposure was evaluated in terms of cell viability, with our data (Figure 3a,b) confirming that transient heating in the 41.5–42.5 °C range does not compromise cell health. Because our exposure conditions are mild and tightly controlled, additional assessment of tissue‐level effects was not required. The calculated cumulative equivalent minutes at 43 °C (CEM43)^[^ ^84^ ^]^ for the worst‐case exposure (≈20 min) remain well below established thresholds for tissue damage.^[^ ^85^ ^]^ Moreover, prior in vivo and cadaveric studies have demonstrated that substantially higher and longer exposures produce no histological injury. In a sheep vertebroplasty model, peak temperatures in the center of the disc reached 41 °C for ≈2.5 min without inducing histological signs of tissue damage.^[^ ^86^ ^]^ Similarly, bipolar RF ablation procedures in human cadaver models achieved inner annular temperatures exceeding 55 °C within 15–18 min of treatment, yet histological analysis confirmed no injury to the NP, annulus fibrosus, or adjacent neural structures.^[^ ^87^ ^]^ In summary, these prior studies involved higher thermal exposures than our protocol, and yet showed no tissue damage. In contrast, our cavitation‐guided ultrasound gelation protocol maintains a tightly controlled temperature range (41.5–42.5 °C) for a significantly shorter duration (1.5–2.5 min), further supporting the thermal safety and clinical feasibility of this approach.

The resulting hydrogels are shown in Figure 6g. To assess the mechanical performance of the hydrogels produced following the cavitation‐guided gelation protocol, an additional sample was produced for rheology (temperature and cavitation data shown in Figure S13, Supporting Information). Alongside this cavitation‐guided ultrasound‐triggered hydrogel sample (US), an incubator‐heated sample (I) was also tested as a control. Both hydrogel materials exhibited G of ≈1.3–1.5 kPa, and shear loss moduli of ≈0.15–0.18 kPa, indicating a good agreement between the different methods used to produce the hydrogels. In addition, we compared the phase angle (δ) and the shear strain at the limit of the linear viscoelastic region (γ), both of which show a good agreement between the ultrasound‐ and incubator‐triggered samples. Overall, these results demonstrated the feasibility of not only using ultrasound to trigger the gelation of injectable biomaterials but also to assess the state of gelation and thus completion of treatment.

#### 2.4.4 Mechanical Testing of Bovine Nucleus Pulposus and Comparison with Hydrogels

The previously reported values of the NP vary depending on the origin of the disc (e.g., human vs animal), the degree of degeneration, the method and parameters used for the test,^[^ ^88^ ^]^ and the position in the body (tail or spine). We chose to test bovine NP using our experimental setup by rheology and directly compare our findings with the values from our biomaterial.

For rheology, each sample was subjected to three frequencies and three amplitude sweep measurements. The G', and G″, G^*^ values for bovine NP were determined to be ≈ 4.4, 1.3, and 4.6 kPa, respectively (Figure 6h). These values were lower than reported values (G^*^ = 7–21 kPa),^[^ ^17^ ^]^ yet higher than the hydrogels formed in this study (G^*^ = 1.5 kPa).

In addition to these observations, the phase angle of the bovine NP is higher than that of our biomaterials, indicating a higher viscous portion, while the shear strain at the limit of the linear viscoelastic region is lower, showing that our hydrogel materials can withstand higher shear deformation.

In summary, our biomaterial presents lower shear and compressive moduli compared to NP tissue. Further optimization could be achieved using double network hydrogels, i.e., hydrogels that consist of two intertwined networks, which have emerged as alternatives to single‐network hydrogels because of the combination of different properties such as Young's modulus and fatigue resistance, originating through the different polymeric components forming the networks.^[^ ^89^ ^]^ Nevertheless, our biomaterial shows promise, as it withstands compressive strains much higher than the target minimum of 25–30% compression strain, which is comparable to physiological strain experienced by the NP, and it shows full recovery upon repeated cycles of high shear strains. Therefore, we have selected the single network hydrogel consisting of alginate, calcium‐loaded liposomes, and microspheres for proof‐of‐concept ex vivo experiments.

### 2.5 Biomechanical Testing of Ex Vivo Functional Spinal Units in Unconfined Compression

**Figure 7:**
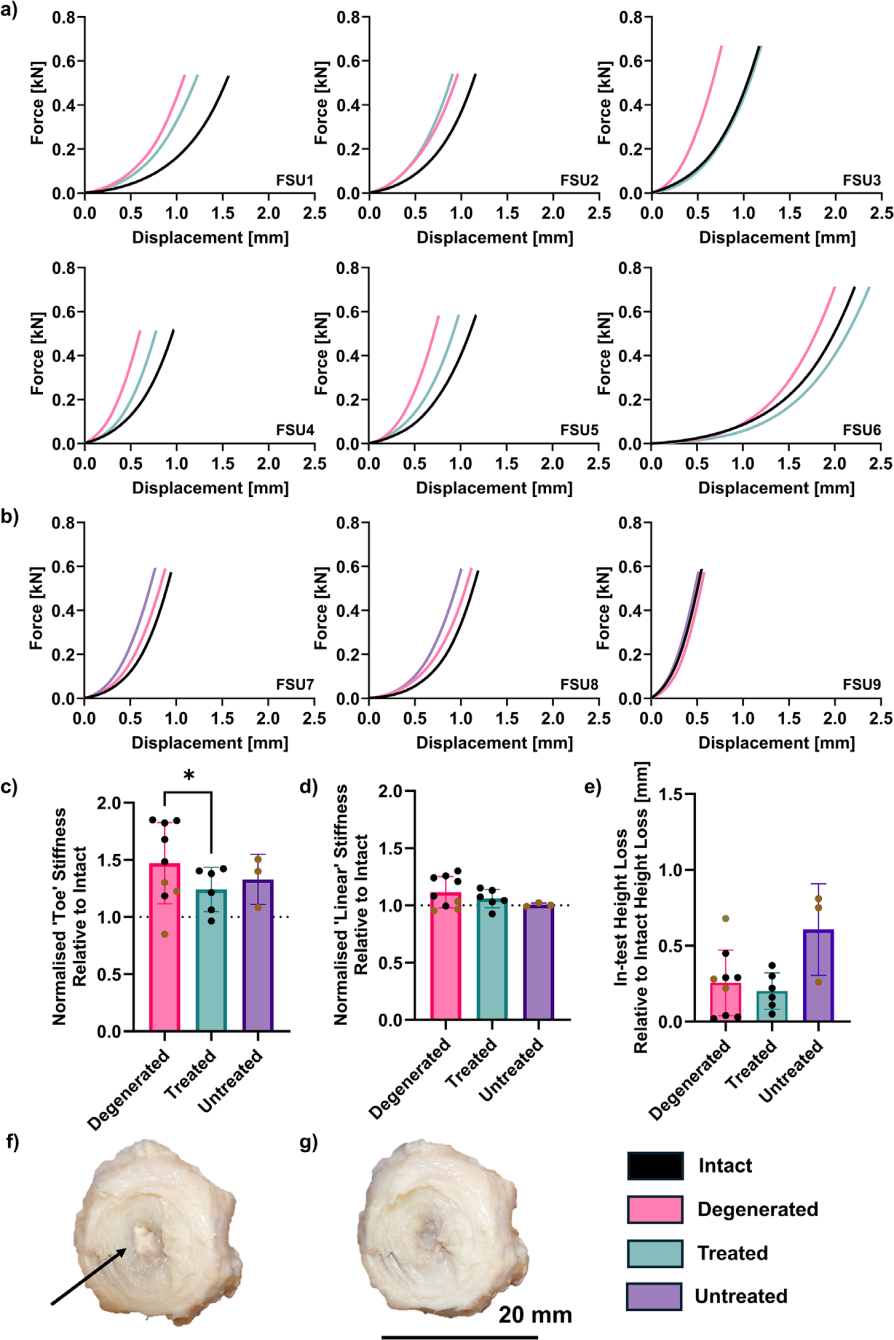
Biomechanical assessment of functional spinal unit performance under uniaxial compression when intact, following degeneration, and with or without treatment (hydrogel injection). Both the treated and untreated groups received ultrasound. a) Mean force‐displacement plots of the treated (*n* = 6 bovine discs) and b) control (*n* = 3 bovine discs) specimens. c) Toe region stiffness (calculated as the gradient between 0.1–0.2 MPa) and d) linear region stiffness (2.2–2.3 MPa) of the treatment and control groups normalized against the mechanical performance of the intact IVDs to provide a more consistent basis for comparison. These ranges were applied consistently across all samples to account for biological variability and ensure comparability. e) Disc height loss relative to intact disc height loss due to compression in the treatment (*n* = 6 bovine discs) and control (*n* = 3 bovine discs) groups. Black datapoints designate the discs in the treatment category that received treatment, while brown datapoints designate the control discs that went untreated. Brown and black data points appear across conditions due to repeated measures of the same treated discs at different time points (e.g., after degeneration (pink) and after treatment (green) or sham (purple)). f) Representative image showing hydrogel integration within the degenerated nucleus pulposus (NP) post‐testing. g) Representative image showing the same tested disc following hydrogel removal. Statistical significance was assessed using mixed effects analysis with the Geisser‐Greenhouse correction plus Tukey's multiple comparisons test, with individual variances computed for each comparison (^*^ *p* < 0.05). Non‐significant differences were not labeled.

The biomechanical performance of six functional spinal units (FSUs) undergoing uniaxial compression was assessed i) when intact, ii) following enzymatic degeneration in the NP, and iii) after hydrogel injection and ultrasound treatment. Three control specimens were also assessed when i) intact, ii) degenerated, and iii) without hydrogel injection (**Figure** **7**). More specifically, these untreated samples were similarly pierced with the injection needle and exposed to the ultrasound procedure; however, no biomaterial was injected.

In all samples, the enzymatic degeneration protocol resulted in increased disc compressive stiffness relative to the intact specimens. In five of the six samples (83%) receiving ultrasound treatment, however, the observed shift in force‐displacement response was partially or fully reversed (Figure 7a; Figure S14, Supporting Information).

This effect was not observed in the three untreated controls (0%) (Figure 7b; Figure S14, Supporting Information), implying that the stiffness changes following degeneration and treatment could not be attributed to either hyperosmotic loading^[^ ^90^ ^]^ or an increase in disc tissue density resulting from a loss of water content following degeneration,^[^ ^91^ ^]^ and that the treatment partially restored disc biomechanics and demonstrated an improvement relative to untreated controls.

Treatment affected the “toe” region (force‐displacement response between 0.1–0.2 MPa) response more substantially than the “linear” region (between 2.2–2.3 MPa). In all of the six treated discs (100%), toe region stiffness was reduced relative to the degenerated samples, while this occurred in none of the three controls (0%), resulting in a significant reduction in stiffness of the treated samples relative to when degenerated (Figure 7c). In five of the six treated discs (83%), the linear region stiffness also reduced following treatment, while this occurred in only one of the three controls (33%) (Figure 7d). However, these effects were non‐significant, as were changes in disc height as a direct result of compressive testing relative to the performance of the intact discs (Figure 7e). Dissection of the treated discs post‐testing revealed that the hydrogel was well integrated within the NP center and difficult to remove (Figure 7f,g; Figure S15, Supporting Information). Variations in the physical consistency of each hydrogel implied differing degrees of hydration.

No implants were herniated from the injection site or in the PBS bath within which mechanical loading was simulated, and no mechanical artifacts suggesting extrusion were present (Figure 7f,g; Figure S15, Supporting Information) ‐ an improvement on many previously proposed solutions. Direct rheological characterization of the hydrogel following in situ gelation within the disc space was not performed in this study. This was due to the relatively small injected volumes (100–150 µL) and the structural integration of the gel with surrounding native disc tissue, which precluded extraction without altering its physical properties. As an alternative, we evaluated the mechanical restoration of the whole disc segment through unconfined compression testing. This approach allowed us to assess the load‐bearing performance of the treated motion segment, providing a functionally relevant measure of the implant's mechanical contribution.

### 2.6 Future Perspectives

While our ex vivo results demonstrate promising material integration and mechanical performance, the long‐term in vivo biocompatibility of the complete material system, comprising sodium alginate, thermosensitive liposomes, calcium ions, and glass microspheres, remains to be established. Alginate, the primary component of our prototype material formulation, has been well‐reported in the literature for its use in biomedical applications. Its well‐established in vitro and in vivo biocompatibility has prompted its use in a variety of tissue engineering and drug delivery contexts, such as in wound healing, with several commercial wound dressings available,^[^ ^92^ ^]^ myocardial,^[^ ^93^ ^]^ and bone tissue engineering.^[^ ^94^ ^]^ Alginate‐based hydrogels have gained attention for their use in IVD degeneration therapies,^[^ ^95^ ^]^ with in vivo studies demonstrating their ability to reduce inflammation and prevent degeneration.^[^ ^96^, ^97^ ^]^

The alginate polymer chains are non‐degradable in mammals, due to the lack of the necessary enzyme.^[^ ^98^ ^]^ However, ionically crosslinked alginate hydrogels are destabilized over time by the exchange of the divalent cations holding the network together with monovalent cations from the surrounding environment.^[^ ^98^ ^]^ The in vivo stability of calcium‐alginate hydrogels has been previously tested in rats, and it has been observed that the mass of the biomaterial decreased only by 17% 3 months after implantation, indicating the long‐term stability of this type of hydrogels.^[^ ^99^ ^]^ While a decrease in the storage modulus of the hydrogel was observed over time, this drop in mechanical properties was less pronounced when calcium carbonate was used instead of calcium chloride.^[^ ^99^ ^]^ In two studies by McDougall and coworkers, calcium alginate hydrogels were used as embolic agents in swine, and it has been shown that calcium alginate hydrogels provided efficient endovascular occlusion for up to 90 days and 6 months.^[^ ^100^, ^101^ ^]^ Several strategies could be implemented to improve the stability of these hydrogels. As the destabilization is due to the displacement of divalent cations by the monovalent cations from the surrounding environment, increasing the number of ionic bridges is expected to prolong the implant's stability. In addition, incorporation of polyphosphate, an anionic polymer, enhances the in vitro stability of the hydrogel through coacervation.^[^ ^102^ ^]^ As a well‐studied biomaterial, alginate has been extensively tested in vitro and in vivo, and its biocompatibility has been demonstrated.^[^ ^100^, ^103^, ^104^ ^]^ Specifically, upon injection of calcium alginate in rats and swine, biocompatibility was confirmed.^[^ ^100^, ^103^ ^]^

Liposomes, which in the current study serve as nanovesicles for on‐demand calcium release, have been previously tested in various biomedical applications, and liposomes based on DPPC or DPSE‐PEG have reached pre‐clinical and clinical trials with no concerns regarding biocompatibility.^[^ ^105^, ^106^ ^]^

Silica‐based (SiO_2_) glass microspheres, a key component in our formulation, also have a well‐documented safety profile in biomedical applications. Notably, silicate bioactive glasses such as Bioglass 45S5 are FDA‐approved for clinical use in bone repair and have shown favorable tissue responses and long‐term safety.^[^ ^107^ ^]^ Moreover, SiO_2_ microspheres exhibit minimal cytotoxicity in numerous in vitro and in vivo studies.^[^ ^108^ ^]^

Together, these data support the suitability of the materials used in the present study; however, we recognize that thorough in vivo biocompatibility assessments of the full composite system are essential. Future studies will therefore focus on detailed safety and biological response evaluations to confirm the clinical translational potential of this ultrasound‐triggered gelation platform.

Beyond the purely mechanical application investigated here, we believe that the therapeutic potential of this biomaterial could be expanded: the biomaterial could act both as a matrix for mechanical support and as a depot for localized and sustained release of compounds of interest. For example, our biomaterial could be used to release bioactive compounds, such as kartogenin for promoting chondrogenesis,^[^ ^109^ ^]^ or growth factors,^[^ ^110^ ^]^ which contribute to tissue repair and regeneration, anti‐inflammatory compounds such as curcumin,^[^ ^111^ ^]^ genetic material such as RNA^[^ ^112^ ^]^ to enhance anti‐apoptosis and provide protective effects, and NP cells.^[^ ^36^ ^]^

While this study focused on in vitro and ex vivo validation of ultrasound triggering, numerous prior investigations have established the feasibility of delivering focused ultrasound through the vertebral column. For example, MRI‐guided FUS has been used for spinal gene delivery and blood‐spinal cord barrier opening in rodents.^[^ ^113^, ^114^ ^]^ Patient‐specific simulations have shown that time‐reversal and phase correction enable accurate targeting of the lumbar IVD with clinically acceptable heating profiles.^[^ ^115^, ^116^ ^]^ Experimental validation using ex vivo human vertebrae further supports the precision of such approaches.^[^ ^117^ ^]^ Additionally, spine‐optimized arrays and interstitial probes have been designed for both diagnostic and thermal applications,^[^ ^115^, ^116^, ^117^, ^118^, ^119^, ^120^ ^]^ highlighting the growing translational readiness of ultrasound‐based spine therapies. These studies collectively support the feasibility of our approach being extended to in vivo systems. Future work will build upon this body of knowledge to optimize delivery through overlying tissues and demonstrate effective targeting of the IVD under in vivo conditions. Future work should also include histological examination of the tissue to determine the biological effects of the treatment and identify potential structural damage exacerbated during an even more physiologically relevant loading scenario.

## 3 Conclusion

This work demonstrates the feasibility of ultrasound‐triggered in situ implant formation for the treatment of IVD degeneration through augmentation, potentially offering a new procedure that would allow the clinician to inject, configure, and solidify the implant on demand at a time of their choosing. Specifically, the sample will remain in a liquid state following injection, and the application of ultrasound will enable the clinician to achieve precise spatiotemporal control over hydrogel formation. Ultrasonic triggering and monitoring of the process can be performed noninvasively to reduce risk and recovery time. Procedural monitoring using the cavitation‐based controller indicated when the implant phase change had occurred, ensuring that procedure duration is guided by in situ conditions rather than a presumed material state following a fixed exposure duration. Key issues to be addressed with future work largely revolve around the injectable biomaterial itself. The compressive elastic modulus of the proof‐of‐concept biomaterial was below that of both bovine and human NP tissues. It was therefore encouraging that partial restoration of biomechanical function was observed in the functional spinal unit testing, but further efforts will be needed to provide a stiffer biomaterial either from the library of options assembled in this project or with alternative engineered biomaterial concepts. The biomaterial design effort would also include considerations for long‐term in situ endurance, alternative components for enhancing ultrasound absorption, and injected volume tracking.

For experimental flexibility, this project employed separate transducers for ultrasound triggering and monitoring, but the use of a single device for all ultrasound functions would be of great value. Therefore, the investigation of a protocol employing existing diagnostic ultrasound systems should be pursued for the benefit of simpler translation and implementation. A broader consideration is that of overall treatment guidance, for which many current IVD therapy concepts employ C‐arm fluoroscopy. Given the vast number of patients that could potentially be treated with the proposed injectable implant technique, there could be a substantial benefit to patient throughput and radiological safety for patients and clinicians alike if ionizing radiation could be eliminated and simpler guidance solutions could be safely and reliably used. Ultrasound guidance with spatially distributed arrays may provide such a capability, and its application for IVD treatments – buoyed by recent advances in array manufacturing and image quality enhancement – merits further near‐term investigation with the aim of greater patient accessibility to the types of minimally invasive procedures described here.

## 4 Experimental Section

### Materials

1,2‐dipalmitoyl‐sn‐glycero‐3‐phosphocholine (16:0 PC, DPPC) and 1,2‐distearoyl‐sn‐glycero‐3‐phosphoethanolamine‐*N*‐{methoxy[poly(ethylene glycol)]‐2000} (ammonium salt) (18:0 PEG2000 PE, DSPE‐PEG2000), 1,2‐distearoyl‐sn‐glycero‐3‐phosphoethanolamine‐*N*‐{biotinyl[poly(ethylene glycol)]‐2000} (DSPE‐PEG2000 biotin), in powder form and solution form (in chloroform 25 mg mL^−1^) were purchased from Avanti Polar Lipids (UK distributor Merck KGaA, Darmstadt, Germany). Sodium hydroxide (reagent grade, ≥ 98%, pellets), deuterium oxide (D_2_O, 99.9% for NMR spectroscopy MagniSolv) and different polysaccharides, as detailed below, were purchased from Sigma–Aldrich Co Ltd (Irvine, UK): poly(galacturonic acid) synthesized via an enzymatic process (PGA_e_, ≥ 90%, molar mass = 25–50 kDa), poly(galacturonic acid) isolated from oranges (PGA_o_, ≥ 90%), alginic acid sodium salt from brown algae (Alg_1_, viscosity 100–200 cP, 1% in water at 20 °C), alginic acid sodium salt from brown algae (Alg_2_, viscosity ≥ 2000 cP, 2% in water at 25 °C), and pectin from apple (Pct, 50–75% esterification, high degree of methylation). Sodium alginate from Macrocystis pyrifera (Alg_3_, viscosity <300 cP, 1% in water, supplied MP Biomedicals), sodium chloride (99.5–100.5%, AnalaR NORMAPUR ACS, Reag. Ph. Eur. analytical reagent), and calcium chloride (≥ 94%, granules, 2–5 mm) were purchased from VWR International Ltd. (Leicestershire, England). Deionized water was obtained from a Triple Red water purification system (Avidity Science, Buckinghamshire, United Kingdom).

In addition, for the calcium concentration experiments in interdigitated and non‐interdigitated liposomes, the glass microsphere preparation, thermometry‐guided in vitro gelation, cavitation‐guided in vitro gelation, and enzymatic degeneration of ex vivo IVD, Fluo‐4, Dulbecco Phosphate‐Buffered Saline, glucose, calcium chloride, sodium chloride, triton x‐100, glass microspheres (SiO_2_, 9–13 µm), ethylenediaminetetraacetic acid (EDTA), SealPlate film, and chondroitinase ABC (cABC) were purchased from Merck (Darmstadt, Germany). Collagenase type II was purchased from ThermoFisher Scientific (Waltham, MA, USA). Deionized water was obtained from a Milli‐Q Ultrapure Water System (Merck, Darmstadt, Germany).

### Thermosensitive Liposome Formulation

Calcium‐loaded liposomes were prepared using an established interdigitation‐fusion vesicle method.^[^ ^65^, ^121^ ^]^ Briefly, a solution containing a 99:1 molar ratio of DPPC and DSPE‐PEG2000 at 25 mg mL^−1^ in chloroform was prepared, and the chloroform was evaporated with a stream of nitrogen. The lipid film was further dried under a vacuum to remove traces of chloroform for at least 3 h. The lipid film was re‐hydrated by adding a 0.4 m calcium chloride solution at 55 °C under constant stirring for 1 h; the final lipid concentration was 20 mg mL^−1^. The concentration of calcium chloride was fixed at 0.4 m, as it was previously shown by Nele et al. that it produces liposomes with the highest calcium loading.^[^ ^65^ ^]^ The liposome solution was extruded 25 times through a 100 nm polycarbonate membrane (Whatman Nuclepore Track‐Etched Membranes diameter 19 mm, Merck, Darmstadt, Germany) and 31 times through a 50 nm polycarbonate membrane (Whatman Nuclepore Track‐Etched Membranes diameter 19 mm, Merck, Darmstadt, Germany). All extrusions were performed at 55 °C. Interdigitation was caused by the addition of ethanol at a final content of 25% v/v.

The interdigitated gels were stored overnight at 2 °C, after which the ethanol was removed by 5 centrifugal washes at 8000 g for 8 min each. Once the ethanol was removed, the interdigitated gel solution was incubated at 55 °C for 150 min, and the resulting solution containing large unilamellar liposomes was extruded 31 times through a 400 nm polycarbonate membrane (Whatman Nuclepore Track‐Etched Membranes diameter 19 mm, Merck, Darmstadt, Germany) at 55 °C. The extrusions were performed using a mini extruder set with a heating block purchased from Avanti Polar Lipids. The excess of calcium ions, i.e., calcium ions that were not loaded in the liposomes, was removed by dialysis against iso‐osmotic buffer (0.6 m NaCl). The purified calcium‐loaded liposome solution was stored at 2 °C until further use. It has been previously shown that liposomal DPPC formulations doped with 1 mol% DSPE‐PEG2000 biotin showed no aggregation propensity and minimal calcium release at 25 °C for 5 days^[^ ^65^ ^]^; thus stability at 2 °C was expected to be prolonged.

For the preliminary data in Figure S2 (Supporting Information), DSPE‐PEG biotin was used instead of DSPE‐PEG to prepare liposomes using the interdigitation‐fusion protocol described above. These liposomes were tested for temperature‐dependent calcium release, as detailed in the relevant section.

To support upscaling efforts, liposomes of the same formulation (99:1 molar ratio of DPPC and DSPE‐PEG2000) were prepared via a modified protocol employing direct extrusion instead of interdigitation‐fusion, and they were used in the cavitation‐guided ultrasound gelation and ex vivo experiments, as detailed below. Protocol details (e.g., lipid concentration, hydration method, temperatures, etc.) were consistent unless specified otherwise. The liposome purification process was also kept the same as previously described. In short, for cavitation‐guided ultrasound gelation, liposomes were prepared using a Genizer Online Liposome Extruder flow cell (Genizer, Irvine, CA, USA) in combination with an LV‐1 Low Volume MICROFLUIDIZER Processor (Analytik Ltd, Cambridge, UK). Liposomes were extruded 7–8 times through 0.4 µm pore‐size membranes (Genizer, Irvine, CA, USA) to ensure a uniform size distribution. In addition, the lipid mixture was sonicated in a heated ultrasonic bath (Digital Heated Ultrasonic Cleaner, Eumax) at 55–60 °C for 20 min to improve dispersion before being extruded through the Genizer flow cell using 0.4 µm pore‐size membranes for consistent liposome sizing. For the proof‐of‐concept ex vivo bovine disc experiment, liposomes were prepared by 31 times extrusion through a 400 nm polycarbonate membrane (Whatman Nuclepore Track‐Etched Membranes diameter 19 mm, Merck, Darmstadt, Germany) at 55 °C, using an Avanti Polar Lipids mini extruder set.

### Dynamic Light Scattering (DLS) for Liposome Sizing

The size of the calcium‐loaded liposomes, prepared by all three methods described above, was determined by dynamic light scattering (DLS). For this experiment, the purified liposome solution was diluted to a concentration of 0.5 mg mL^−1^ in iso‐osmotic buffer (0.6 m NaCl), unless stated otherwise. The measurements were performed using a Zetasizer Nano ZS from Malvern Instruments Ltd. (Malvern, UK). The DLS measurements were performed at 25 °C, and the scattered light was collected at a backscatter angle of 173°. Each sample was measured three times. The DLS curves show the normalized intensity, number, and volume as a function of the hydrodynamic diameter. The data analysis was performed using Zetasizer software (v. 8.02) from Malvern Panalytical.

### Cryogenic Transmission Electron Microscopy

The liposome solution and its mixture with Alg_1_ (1.5% w/v polymer and 15mm entrapped Ca^2+^ in liposomes) were imaged via cryogenic transmission electron microscopy (cryo‐TEM). For sample preparation, 5 µL of sample was applied to a freshly glow‐discharged lacey carbon grid (EM Resolutions, UK) and plunge‐frozen using a Leica GP2 plunge‐freezer. Grids were imaged using a JEOL 2100Plus FS with a Gatan OveView camera. The analysis was performed using ImageJ (1.53p, Wayne Rasband and contributors, National Institute of Health, USA).^[^ ^122^ ^]^

### Temperature‐Dependent Calcium Release of Interdigitated Liposomes

For this experiment, 50 µL of liposome sample in 500 µL tubes was immersed in a water bath set at the desired temperature. The following temperatures were targeted: 30, 35, 37, 39, 40, 41, 42, 43, 44, 45, and 49 °C. Samples were equilibrated for 15 min at each temperature with a thermocouple placed inside the test tube to monitor the temperature for the whole incubation time. At the end of the incubation time, samples were cooled to 20 °C, and the released calcium was measured using the *o*‐cresophtalein complexone (*o*‐CPC) assay, as previously described.^[^ ^65^ ^]^ For this, samples were diluted to have a total encapsulated calcium concentration of 2 mm to be in the linear range of the *o*‐CPC assay. A standard curve was constructed using unloaded liposomes at a matching particle concentration and spiked calcium. The particle concentration of calcium‐loaded and unloaded liposomes was measured by nanoparticle tracking analysis (NTA); for this, samples were diluted to a concentration of 10^8^–10^9^ particles mL^−1^ in iso‐osmotic buffer and three 60‐s videos were acquired using a NanoSight NS300 at a camera level of 13. The videos were then analyzed using NTA V3.0 software with a detection threshold of 5 to obtain the particle concentration.

### Calcium Concentration in Interdigitated and Non‐Interdigitated Liposomes

The calcium concentration in the liposome samples was measured fluorometrically with Fluo‐4 (Merck, Darmstadt, Germany). Because of the high calcium concentrations used during liposome production, liposomes prepared in the absence of calcium were no longer deemed suitable to use as a blank. The matrix was, therefore, treated as an unknown, and the measurements were calibrated by standard addition. Each sample (1 µL) was diluted 1:10.000 with an osmolality‐matched solution of 0.9 m glucose in Dulbecco Phosphate‐Buffered Saline, and Fluo‐4 was added such that the fluorescence response was linear. The sample was then split into 4 100 µL aliquots, and each aliquot was immediately spiked with 0, 5, 10, or 15 µL of calcium standard (26.7 µm CaCl_2_ in 0.9 m glucose in DPBS) to obtain calcium concentrations of standard corresponding to the original undiluted sample concentrations of 0, 13.3, 26.7, and 40.0 mm, respectively. After incubating for 10 min at room temperature, fluorescence emission was measured in a plate reader (Omega FluoStar, BMG LABTECH GmbH, Ortenberg, Germany; excitation 490 ± 10 nm, emission 520), and the calcium concentration in the sample was estimated as minus the x‐intercept of the linear regression of fluorescence against concentration of standard. The following samples were tested: 1) original liposomes, e.g., as prepared, 2) with the addition of 1% Triton X‐100, incubated for 10 min at 60 °C, and cooled to room temperature for 1 h before fluorescence emission was measured.

### Physicochemical Characterization of Polymers—Proton Nuclear Magnetic Resonance (^1^H NMR) Spectroscopy

The NMR samples were prepared by dissolving the alginate polymers in D_2_O at concentrations of 5‐10 mg mL^−1^. The solutions were analyzed by using a 500 MHz Avance Bruker NMR spectrometer from Bruker (Bruker, UK Ltd., Coventry, UK), and the data analysis was performed on MestReNova (version 15.0.1‐35756, 2024 Mestrelab Research S.L.).

### Physicochemical Characterization of Polymers—Gel Permeation Chromatography (GPC)

The GPC samples were prepared by dissolving the alginate polymers in 0.1 M NaNO_3_ aqueous solution at concentrations between 1.0 and 3.0 mg mL^−1^. Prior to the measurement, the polymer solutions were filtered through 0.45 Nylon filters. The samples were analyzed using an Agilent Infinity II instrument equipped with the following detectors: differential refractive index, viscometry, and dual‐angle light scattering. The system was equipped with two Agilent PLaquagel‐OH Mixed M columns (300 × 7.5 mm) and an Agilent PLaquagel guard column (50 × 7.5 mm). The GPC solvent, namely 0.1 m NaNO_3_ aqueous solution, was pumped through the system at a flow rate of 1 mL min^−1^ at 35 °C. Polyethylene glycol/oxide Easivials, purchased from Agilent Technologies UK Ltd, were used to calibrate the instrument by applying a third‐order universal calibration from refractive index and viscometry data between 0.610 and 474 kDa. The analysis was performed using Agilent GPC/SEC software.

### Preparation of Hydrogel Precursor Solution

Sodium alginate solutions were prepared by dissolution of the solid polymer at different concentrations in deionized water without further pH adjustment. Poly(galacturonic acid) sodium salt solutions were prepared by dissolution of the polymer in 10 mm NaCl, followed by pH adjustment to ≈6.4 to 6.5 by using sodium hydroxide solution, following a procedure previously reported in the literature.^[^ ^72^ ^]^ Pectin solutions were prepared by dissolution of the polymer in 10 mm NaCl, followed by heating to 40 °C to obtain a slightly cloudy orange solution. The alginate/liposome formulation was prepared by mixing the polymer and the liposome solutions at a volume ratio of 1:1, thus leading to a final entrapped Ca^2+^ concentration of 15 mm. The initial alginate concentration was varied from 4, to 3, to 2% w/v, thus leading to a final alginate concentration of 2, 1.5, and 1% w/v, respectively. For the polygalacturonate/liposome formulations, the initial PGA concentration was kept constant at 2 w/v%, and the volume ratio of the polymer to liposome solution was varied from 1:3, to 1:1, to 3:1, thus leading to final polymer concentrations of 0.5, 1, and 1.5% w/v, while simultaneously changing the entrapped Ca^2+^ concentration from 22.5, to 15, to 7.5 mm. The Pct/liposome formulation was prepared by mixing a 2% w/v polymer solution and the liposome precursor solutions at a volume ratio of 1:1, leading to a final entrapped Ca^2+^ concentration of 15 mm.

### Preparation of Incubator‐Heated Hydrogels

To produce hydrogels of cylindrical shape for rheological tests, the precursor solution, i.e., the solution containing the anionic polysaccharide and calcium‐loaded liposomes, was heated in an incubator above the release temperature of the liposomes (41 °C, which is the melting temperature of the DPPC lipids) to promote calcium release and thus gelation. To cast the hydrogels at a cylindrical shape and specific dimensions required for rheological measurements, positive molds were 3D printed in‐house using a Prusa SL1S printer and then used to fabricate the negative replica using EcoFlex. The final Ecoflex molds were used to cast the hydrogels in cylindrical concavities of 8 mm diameter and 1 mm height. The molds were placed in an incubator set to 45.5 °C for 10 min to promote gelation. The temperature of the incubator was set to a higher value than the target value of 41.0 °C to allow the sample to be heated to 41.0 °C and thus promote sufficient calcium release and gelation. Using thermometry (RS PRO Wired Digital Thermometer, equipped with a thermocouple and UKAS calibration, RS components, Corby, UK), it was identified that the temperature of the sample stabilized around 41.0 °C after 10 min of incubation. After incubation, the gels were visually inspected for gelation by removal from the mold before being subjected to additional tests.

### Preparation of Bovine NP for Rheology

Bovine discs were selected as a model due to their similarities in size and mechanical response with human tissue and the scarcity, cost, and mechanical variability of the latter. This also ensured greater control over the extent of tissue degeneration. Bovine tails were sourced from a local abattoir and dissected to remove all soft tissue. To preserve tissue hydration, tissues were periodically sprayed with DPBS. A band saw was then used to cut the first two caudal vertebral bodies at the mid‐transverse plane, yielding a bone‐disc‐bone structure or functional spinal unit (FSU). For three bovine FSUs, the vertebrae were carefully removed from the IVD using a scalpel. While frozen, the NP was cut out of the IVD using an 8 mm diameter biopsy punch and subsequently cut into 1 mm thick slices using a custom‐designed tool, which was chilled before use at −20 °C for 30 min. All processing was performed at 4 °C. Four slices per bovine IVD were selected for the rheological measurements.

### Rheology

Rheological measurements were performed using an AntonPaar modular compact rheometer MCR203, equipped with a parallel measuring plate. The PP08 plate was used for all rheological measurements on the hydrogels, while the PP25 was used for the flow‐sweep measurements on the hydrogel precursor. The data were analyzed using AntonPaar RheoCompass V1.23.378 software. The following experiments were performed on three liposome batches (N = 3), and three samples were tested per batch (*n* = 3), unless stated otherwise.

### Rheology—Flow‐Sweep Measurements (Viscosity vs Shear Rate)

Flow‐sweep measurements were performed to investigate the shear‐thinning properties of the samples before gelation, which indicates their injectability. The viscosity (η, Pa s) was measured at 25 °C as a function of the shear rate, which was varied from 0.01 to 100 s^‒1^. Each sample was tested three times.

### Rheology—Amplitude‐Sweep Measurements

Amplitude‐sweep measurements were performed to determine the LVR of each sample, i.e., the area of amplitude values under the application of shear deformation that did not alter the rheological properties of the material. For this experiment, Gˊ and G˝ were recorded at 37 °C at a fixed angular frequency (ω) of 1 rad s^−1^, while the shear strain was varied from 0.1% to 100%. Each sample was tested three times. The strain value defining the LVR was calculated as the intersection between the line fit to the plateau and the drop‐off regions.^[^ ^123^ ^]^

### Rheology—Frequency‐Sweep Measurements

Frequency‐sweep measurements were performed to investigate the variation of the Gˊ and G˝ moduli as a function of the angular frequency. The experiment was performed at 37 °C, by varying the angular frequency from 0.1 to 100 rad s^‒1^ at a fixed shear strain of 0.1%, which was previously determined by amplitude‐sweep measurements to fall within the LVR of the biomaterial. Each sample was tested three times. The hydrogels produced via the thermometry‐guided method, Figure 5 and Figure S11 (Supporting Information), were tested at a fixed shear strain of 0.5%, which also falls within the LVR of the biomaterial (see Figure S11b, Supporting Information).

### Rheology—Strain‐Recovery Measurements

Strain‐recovery measurements were performed to investigate the recovery of the Gˊ and G˝ moduli upon application of alternating low and high shear strain values at 37 °C. The low strain value was kept at 0.1%, which was previously determined by amplitude‐sweep measurements to fall within the LVR of the material. The high strain values were aimed at 10% and 100%. Three cycles of 60 s at low shear strain and three cycles of 60 s at high shear strain were performed in an alternating manner at 37 °C. The angular frequency was kept constant at 1 rad s^‒1^.

### Rheology—Temperature Ramp Measurements

Temperature ramp measurements were performed to investigate the gelation temperature and values of shear storage and shear loss moduli. This experiment was performed at a heating rate of 0.15 °C min^−1^ from 40 to 50 °C at a shear strain value of 0.1% and angular frequency at 1 rad s^−1^.

### Unconfined Compression Testing

Ultrasonically triggered hydrogel samples were cast as described in the relevant section and then subjected to unconfined compression tests using a TA Electroforce 3200 equipped with a 4 N load cell (New Castle, Delaware, USA). The values were normalized to 0.008 N initial force, and Young's modulus (E) was calculated in the linear region of the graph, between 5% and 10% compressive strain, within the linear region of the material. The tangent moduli (E_t_) were calculated at higher compressive strains, as indicated in the relevant figure.

### Glass Microsphere Preparation

Before hydrogel incorporation, glass microspheres (SiO_2_, 9–13 µm, Merck, Darmstadt, Germany) were purified to remove divalent cations (e.g., Ca^2+^) that might induce premature gelation. For purification, 2 g of glass spheres were suspended in 50 mL of 100 mm ethylenediaminetetraacetic acid (EDTA, pH 8, Merck, Darmstadt, Germany) in MilliQ and rotated overnight on a roller mixer (SRT6, Stuart, Stone, UK). The EDTA was removed by vacuum filtering. The spheres were then resuspended in fresh EDTA and shaken (IKA KS 130 basic, Staufen, Germany) for 90 min, repeating this step once. Spheres were then washed twice with MilliQ and finally resuspended in 7 mL of MilliQ. Solutions were snap frozen in liquid nitrogen and freeze‐dried (VirTis adVantage Plus, Warminster, Pennsylvania, USA) at −20 °C (shelf), −80 °C (condenser), vacuum <0.6 mbar, for 72 h.

### In Vitro Biocompatibility Tests—Cell Culture

H9 embryonic stem cells (hESCs, WiCell, WA09, passage 36) were cultured feeder‐free on hES qualified Corning Matrigel according to manufacturer instructions, and medium changes were performed daily with fresh mTESR Plus (StemCell Technologies, Cat#100‐0276). Cells were passaged using 0.5 mM EDTA (Invitrogen EDTA (0.5 m), pH 8.0, RNase‐free), diluted 1:1000 in PBS pH 7.4 without calcium and magnesium ions, and reseeded as small clumps into 24‐well plates for biocompatibility testing. Primary human cardiac fibroblasts (hFIBs, from PromoCell, isolated from the ventricles of the adult heart, passage 7) were cultured in 6‐well plates (Corning Costar Clear TC‐treated Multiple Well Plates, (Cat#3516)) in fibroblast growth media 3 (from PromoCell) and 1:100 PenStrep (Thermo Fisher, Cat#15140122). Medium changes were performed every 3 days with 3 mL of fresh media. Human mesenchymal stem cells (hMSCs, passage 4) were cultured in 6‐well plates (Corning Costar Clear TC‐treated Multiple Well Plates) in hMSC media DMEM/F12+HEPES (Thermo Fisher, Cat#11 330 032), Glutamax (Thermo Fisher, Cat.# 35050038, 10% fetal bovine serum (FBS), and 1:100 PenStrep. Medium changes were performed every 3 days with 3 mL of media. Components and items used for cell culture experiments, apart from those explicitly specified, were purchased from ThermoFisher Scientific, Waltham, MA, USA. All cell culture was performed at 37 °C, 5% CO_2_ in humidified incubators. Cells were routinely tested for Mycoplasma. For metabolic and viability assays, hESCs were plated in an area ratio of 1:12 into 24‐well plates. For hFIBs, 40 000 cells were plated into each well of a 24‐well plate (Corning Costar 24‐well clear TC‐treated Well plate (Cat#3524). For hMSCs, 14 000 cells were plated into each well of a 24‐well plate.

### In Vitro Biocompatibility Tests—Material Preparation and Cell Exposure

The cells were exposed to different conditions for 24 h, prior to testing the biocompatibility via metabolic and live‐dead assays, as described in more detail below. The conditions used were: i) cells only (negative control), ii) hydrogel precursor (1.5% wt/v Alg_1_ solution seeded with calcium‐loaded liposomes (10–15 mm calcium chloride) containing 6% w/v microspheres), iii) hydrogel (1.5% wt/v Alg_1_ solution seeded with calcium‐loaded liposomes (10–15 mm calcium chloride) containing 6% w/v microspheres), and iv) Triton X‐100 (positive control). Transwell inserts with a 6.5 mm diameter and 0.4 µm pore polyester membrane (Scientific Laboratory Supplies Ltd, Nottingham, UK) were used during the experiments. For all treatment groups, the respective materials (hydrogel, hydrogel precursor, or Triton X‐100) were added to the apical compartment of the transwell inserts, and the cells were seeded at the basolateral side. For the “hydrogel” condition, the material was directly crosslinked inside the transwell inserts in a well plate without cells by placing the transwells containing precursor solution in an incubator set at 50 °C for 13 min to promote sufficient gelation. In addition to the conditions described above, cells were exposed to transient heating to evaluate its effect on viability. To this end, the well plate was placed in an incubator set to 45 °C for 15 min, allowing the cells to reach a target temperature of 42 °C. Under these conditions, the temperature stabilized between 42–43 °C for ≈2.5 min, replicating the thermal profile observed during the ultrasound experiments. This heating protocol was determined based on incubator temperature curves obtained using a thermocouple‐equipped RS PRO Wired Digital Thermometer (UKAS‐calibrated, RS components, Corby, UK).

### In Vitro Biocompatibility Tests—Metabolic Assay

A CCK‐8 assay (Abcam Ltd, Cambridge, UK) was performed after 24 h of incubation according to manufacturer instructions and absorbance at 450 nm was measured after 1 h of incubation using a Spectramax M5 plate reader spectrophotometer (Molecular Devices, Berkshire, UK).

### In Vitro Biocompatibility Tests—Viability Staining

LIVE/DEAD staining was performed using a LIVE/DEAD Cell imaging kit (Thermo Fisher, Cat#R37601) according to manufacturer instructions. In brief, cells were incubated with a 50:50 mixture of media: staining buffer for 15 min at room temperature before imaging. Cells were imaged using a Nikon Eclipse Ti2 inverted microscope (Nikon Europe B.V., Amstelveen, The Netherlands). For each well:

(5) $$\%\;\mathrm{Live}=\frac{\mathrm{Live}}{\mathrm{Live}+\mathrm{Dead}}*100$$

Per cell line, each condition (control, hydrogel, precursor, and death control) was measured in triplicate wells.

### In Vitro Biocompatibility Tests—Quantifications

Quantifications were performed using the cell counter plugin in Fiji (ImageJ 1.54f, Wayne Rasband and contributors, National Institute of Health, USA). Data are shown as individual wells with mean ± S.D. For the live count of hESCs, the average area of individual cells was measured (≈151 ± 45 µm^2^) and extrapolated to the colony size area. For the CCK‐8 assay, the data were normalised against the control (no exposure), and negative values have been capped to 0.

### Attenuation Measurement and Data Processing

Estimation of gel precursor ultrasound attenuation was performed using a through‐transmission technique.^[^ ^124^ ^]^ The primary system components were a spherically focused source for generating a broadband field, a needle hydrophone for pressure field measurement, and a sample holder containing the material to be analyzed (Table S1, Supporting Information). Both transducers were submerged 12 cm apart in deionized, degassed water at 37.5 °C, and the sample holder was placed between the devices with the sample front face located at the source's geometric focus. To produce short‐duration, broad‐bandwidth test signals, the source transducer was driven by a pulser‐receiver. These signals were measured with the hydrophone alternately with water and hydrogel precursor biomaterial in the container.

Attenuation (α_*s*_) was estimated based on the ratio of the transmitting voltage response for the sample (TVR_s_) to the degassed water control (TVR_w_):

(6) $$\alpha_{s}=-\frac{\ln\left(\frac{TVR_{s}}{TVR_{w}}\right)}{d_{s}}+\alpha_{w}$$

where *d_s_* is the sample thickness, α_*w*_ is the attenuation in water (0.00025*f^2^*) in units of (Np cm^−1^ MHz^−1^,^[^ ^125^ ^]^ *f* is the frequency in MHz, and TVR is the received pressure spectrum normalized by the transducer drive spectrum. The final sample attenuation was calculated by averaging the TVR ratio in Equation (6) over five pressure field scan points spanning 0.6 mm centered in the main transmission lobe. The frequency range retained for these measurements was defined based on a measurement signal‐to‐noise ratio ≥ 30. To estimate the acoustic attenuation at therapeutic transducer frequencies, e.g., 0.95–1.25 MHz, the data for *α*_s_ was fitted with a power law expression:^[^ ^126^ ^]^

(7) $$\alpha_{s}=a_{0}f^{n}$$

where *a*_0_ and *n* were obtained by least‐squares fitting of the measured data.

Measurements of attenuation and sound speed in bovine discs were carried out using similar procedures, except with the transmitting and receiving transducers mounted on a set of calipers.^[^ ^127^ ^]^

### Ultrasound Protocol and Apparatus Development

**Figure 8:**
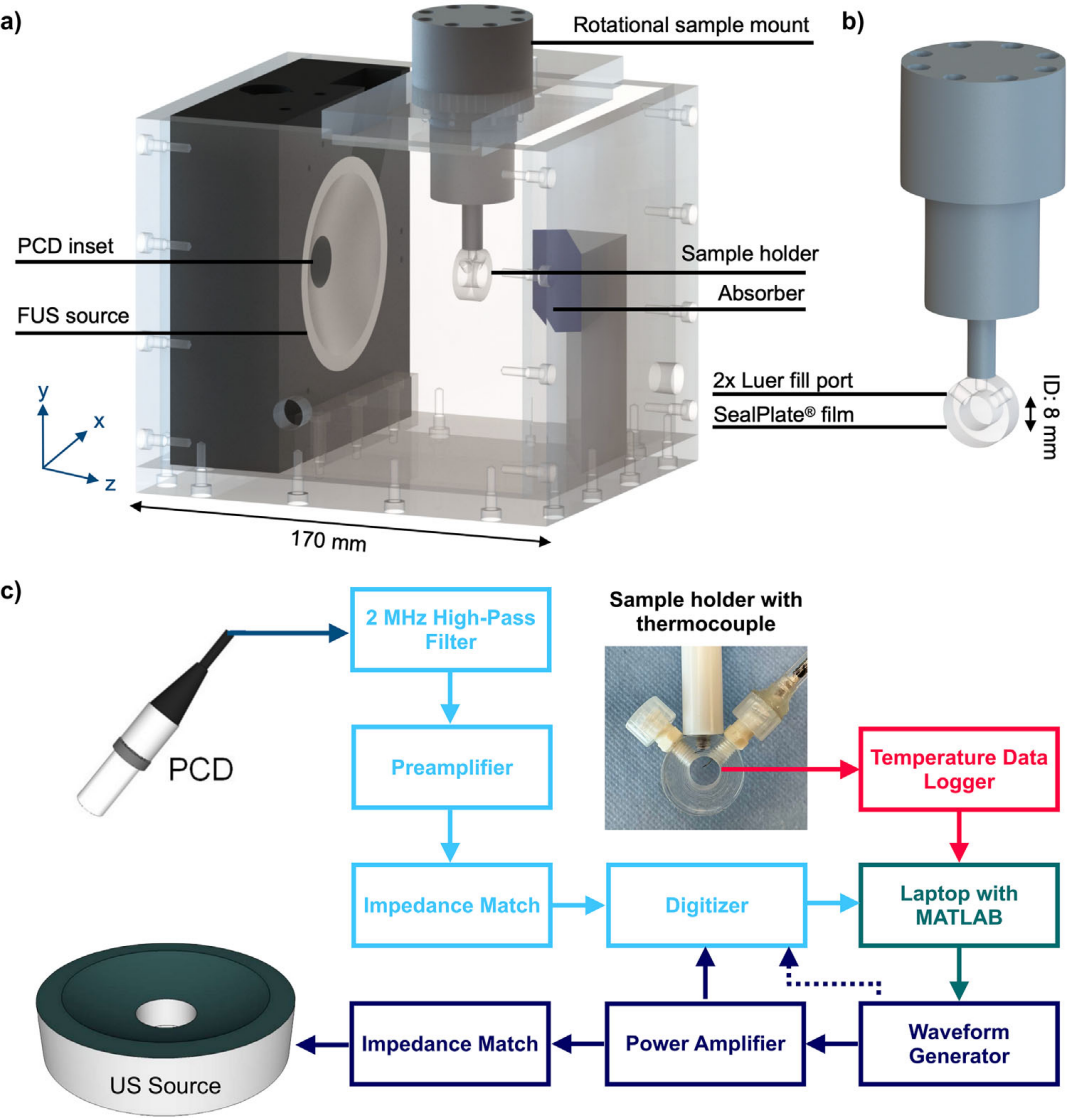
Ultrasound‐triggered gelation test apparatus. a) Chamber outfitted with a focused ultrasound (FUS) source and PCD transducers, a rotational sample mount to ensure perpendicular alignment to the sound field, and a terminating absorber (blue). b) Sample mount and the sample holder, consisting of a Perspex cylinder fitted with 2 Luer ports and sealed front and back by SealPlate® film. c) Instrumentation for PCD and thermometry signal conditioning and recording.

A custom apparatus (**Figure** **8**a) was designed for preliminary optimization, featuring a therapeutic transducer (Sonic Concepts, H102, 1.1 MHz center frequency, radius of curvature (ROC) of 63.2 mm, Bothell, WA, USA), and a passive cavitation detector (PCD, 7.5 MHz center frequency / 0.5″, Olympus NDT, Waltham, MA, USA) in a right‐angle mirror configuration integrated at the ultrasound source base. The 1.1 MHz transducer, with a broad bandwidth (≈0.9–1.3 MHz), enabled frequency studies without changing devices and was appropriate for propagation and heating across several cm of tissue. The drive waveform was provided by a signal generator (Agilent Technologies, 33250A, Santa Clara, CA, USA), amplified (E&I 325LA, Rochester, NY, USA), and directed through the transducer's impedance matching network.

The PCD, with its 2–8 MHz bandwidth, enabled effective cavitation monitoring in hydrogel samples. The PCD signal passed through a 2 MHz high‐pass filter (Allen Avionics Inc., F5081‐2P0, River Grove, IL, USA), was amplified (SR445A, Stanford Research Systems, Sunnyvale, CA, USA), and captured via a USB oscilloscope terminated at 50 Ohms (HS6, TiePie Engineering, Sneek, The Netherlands). MATLAB was used to analyze the PCD traces, generating power spectra to quantify spectral components: harmonics (integer multiples of the transducer center frequency f_0_), ultraharmonics (odd multiples of f_0_/2), and broadband noise, indicative of inertial cavitation, calculated by removing harmonics and ultraharmonics. The tank temperature was stabilized at 36.0 ± 0.5 °C by heating degassed deionized water, with the tank placed in an insulated water bath at 41 °C maintained by a circulating heater (T100, Grant Instruments, Shepreth, UK).

### Thermometry‐Guided In Vitro Gelation

For ultrasound heating tests, candidate biomaterials were injected into a Perspex cylinder with two Luer connectors—one for sample input and the other to release macrobubbles (Figure 8). Samples equilibrated to the water bath temperature of 36.0 ± 0.5 °C (≈5–10 min). The cylinder ends were sealed with 0.07 mm adhesive membranes (SealPlate film, Merck, Darmstadt, Germany) to maintain acoustic and optical transparency. After filling the holder with gel precursor through an 18‐gauge needle (within the needle size range used clinically for radiofrequency ablation for chronic low back pain),^[^ ^128^ ^]^ a thermocouple (type‐T, HYP0‐33‐1‐T, Omega, Stamford, Connecticut, USA) was inserted through a modified Luer connector and positioned securely. A second thermocouple (TC direct, 0.25 mm diameter, type T, 406‐596, Uxbridge, UK) was placed in the ultrasound tank corner to register baseline temperature. Temperature was logged (PicoTechnologies, TC‐08, St Neots, UK). Thermocouples, due to viscous heating, can cause temperature artifacts upon ultrasound exposure. Thus, probe sizes were chosen to minimize this effect (d <λ/20 as per Fry and Fry, 1954;^[^ ^129^ ^]^ d <λ/5 per Hynynen and Edwards, 1989),^[^ ^130^ ^]^ though small sizes may still introduce significant artifacts. An extrapolation method similar to Parker (1983)^[^ ^131^ ^]^ and Maruvada et al. (2012)^[^ ^132^ ^]^ was applied: a time window (2–10 s after ultrasound cessation) was used for a cubic polynomial fit of the thermal decay curve, then extrapolated to determine the temperature rise excluding thermocouple effects. Processing was done using MATLAB.

To automate heating, custom MATLAB code toggled the function generator output via thermocouple feedback (USB serial communication). Temperature limits were set at 41.5 °C (lower) and 42.5 °C (upper) to release Ca^2+^ from liposomes with a phase change at 41.0 °C. The function generator was active until reaching the upper limit; cooling reactivated it once the temperature dropped below the lower limit. This cycle continued for 5 min, maintaining the temperature above the minimum. After 5 min, the ultrasound was automatically turned off, and cooling was recorded. The environmental baseline was recorded for 20 s before ultrasound initiation, with temperature recording manually stopped after 8 min.

### Cavitation‐Guided In Vitro Gelation

To simplify clinical application and leverage cavitation as an indicator of gelation, automated cavitation‐based guidance was implemented instead of thermometry. Custom MATLAB code was updated to perform real‐time acoustic signal processing, specifically monitoring broadband emissions as an indicator of treatment progress. A cavitation threshold was set based on the noise floor recorded during a 20‐s baseline, with an added tolerance from preliminary data. The treatment protocol began with an 80‐s ultrasound application, followed by real‐time cavitation monitoring. The median broadband emissions over the last 50 samples were continuously compared to the threshold: if exceeded, ultrasound cycles (9 s on, 7 s off) continued; otherwise, ultrasound was halted, marking gelation completion. For experimental validation, 3% wt/v alginate with 12% wt/v glass microspheres was prepared, mixed 1:1 with liposomes in 0.6 m NaCl to achieve a 1.5% wt/v polymer concentration and 6% w/v microsphere concentration, and injected in 8 mm inner diameter sample holders using an 18‐gauge needle. Temperature data was passively collected in select samples to confirm the safety of the cavitation‐guided protocol.

### Enzymatic Degeneration of Ex Vivo IVDs

At the point of degeneration, each FSU was incubated for 30 min at 37 °C, then 100 µL of collagenase type II (2 mg mL^−1^; ThermoFisher Scientific, Waltham, MA, USA) and chondroitinase ABC (cABC) (2 U mL^−1^; Merck, Darmstadt, Germany) in DPBS was injected into the center of the FSU's NP using a 29‐gauge needle, followed by a 4‐h incubation at 37 °C.^[^ ^133^ ^]^ A cut 18‐gauge needle was then inserted at the injection site, and the discs were placed in 4 L of DPBS at 4 °C overnight to inactivate and disperse the enzymes.

### Proof‐of‐Concept Experiment

**Figure 9:**
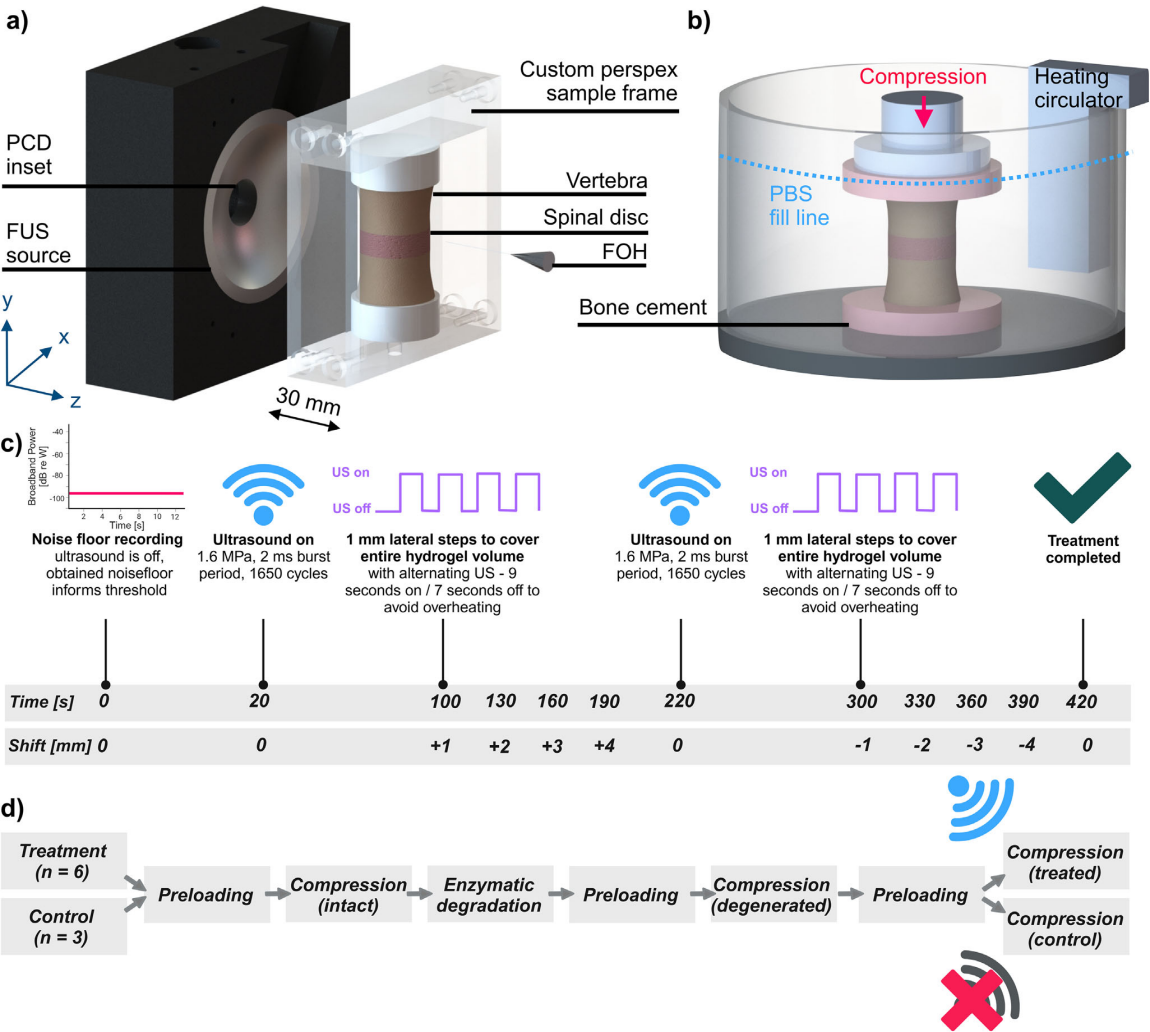
Ultrasound‐triggered ex vivo bovine set‐up, with a FUS source and a PCD, a custom‐made rig to hold a functional spinal unit consisting of two vertebrae and the IVD, which is aligned to the FUS source focus with a fiber optic hydrophone (FOH). b) Test set‐up enabling the biomechanical assessment of intact, degenerated, and treated ex vivo bovine specimen performance under uniaxial compression in DPBS at 37 °C. c) The ex vivo bovine focused ultrasound protocol involved a 20‐s baseline recording followed by 80 s of ultrasound at x = 0 mm (center of the disc and hydrogel injection site). After these 100 s, the ultrasound was scanned laterally across the transverse plane in 1 mm steps, with 9 s on and 7 s off, for 30 s per position to prevent overheating. After a 4 mm displacement in one direction, the ultrasound was returned to the center to reheat before scanning in the opposite direction using the same on/off sequence. Treatment concluded after the 30‐s exposure of the final position. Created in BioRender. Kaesbauer, S. (2025) https://BioRender.com/j12t503. d) Biomechanical assessment protocol of intact, degenerated, and treated disc performance. Created in BioRender. Kaesbauer, S. (2025) https://BioRender.com/h29p001.

A custom rig was developed to position the FSUs at the ultrasound focus. In a degassed water tank, the FSUs and custom rig were placed within an acoustically transparent plastic bag filled with degassed DPBS. This rig was mounted on a custom automatic 3D positioning system (UMS 3, Precision Acoustics, Dorchester, UK) while the ultrasound source and PCD block remained stationary in the tank (**Figure** **9**a). Testing was conducted at 36.5 ± 0.5 °C, maintained by a circulating heater (T100, Grant Instruments, Shepreth, UK). The ultrasound focus was verified using a fiber optic hydrophone (FOH) system (125 µm diameter; Precision Acoustics, Dorchester, UK) mounted on an XYZ‐positioning system (UMS 3, Precision Acoustics, Dorchester, UK). The FOH was carefully realigned, and the FSU was positioned based on the time‐of‐flight from the source to the FOH, corresponding to the transducer's ROC plus the disc radius at the injection site, positioning the ultrasound focus on the center of the IVD (Figure 9a). Ultrasound parameters for this alignment were set at 1.1 MHz, 2 MPa PNP, with a 10 ms burst period and 11 cycles.

Hydrogel (1.5% wt/v low‐viscosity sodium alginate Alg_1_, 6% wt/v microspheres mixed 1:1 with Ca^2+^‐loaded liposomes with final [NaCl] = 0.3 M) was injected through an 18‐gauge needle at the designated injection site (defined as the needle puncture hole created during enzyme injection to achieve degeneration) until backpressure indicated adequate filling. To prevent pre‐treatment gel leakage, discs were wrapped in parafilm (Merck, Darmstadt, Germany), and the injection site was marked for alignment with the FOH. The ultrasound (0.95 MHz, 1.6 MPa PNP, 2 ms burst period, 1650 cycles) treatment protocol included a 20‐s baseline noise recording, followed by 80 s of ultrasound at the disc center, then lateral scans in 1 mm increments every 30 s up to 4 mm in each direction, with a ‘9 s on, 7 s off’ sequence (Figure 9c). This approach mitigated errors in targeting the hydrogel‐filled area. After treatment, parafilm was removed, and discs were wrapped in DPBS‐soaked tissue for hydration, then stored at 4 °C until further use.

### Compressive Testing of Treated Functional Spinal Units

During daily activity, intradiscal pressure in the largest human lumbar disc can range from 0.1 MPa when lying prone to 2.3 MPa during physical activity, such as lifting a 20 kg weight with a round flexed back.^[^ ^134^ ^]^ Consequently, a cyclic loading protocol applying this stress range was developed to assess the effect of the implant on native disc biomechanical performance under physiological loading. Nine FSUs were potted using PMMA via a previously reported method.^[^ ^135^ ^]^ The cross‐sectional area of each disc was recorded using calipers to convert stress into compressive force, where the disc anteroposterior and mediolateral diameters were measured three times each, and a circular disc geometry was assumed. Maximum loads and loading rates were based on established intradiscal pressures within human lumbar IVDs, scaled by a factor of 0.66 to account for the discrepancy between measured intradiscal pressure and the applied stress required to generate this (Table S2, Supporting Information).^[^ ^136^ ^]^ All FSUs were tested non‐destructively three times: first, while intact; second, following enzymatically‐induced degeneration; and finally, after receiving either treatment with the injectable biomaterial or a control treatment without injection (Figure 9d).

Cyclic, uniaxial compression tests were performed on FSUs using a screw‐driven materials testing machine equipped with a 10 kN load cell (5578, Instron, Massachusetts, USA) (Figure 9b). All FSUs were preloaded to normalize tissue hydration by submerging them in DPBS and applying a 0.1 MPa (≈25 N) uniaxial load at 4 °C for 16 h, followed by 1 h at 37 °C. FSUs were then subjected to four preconditioning cycles of uniaxial compression from 0.1 MPa (≈25 N) to 2.3 MPa (≈500–700 N) at 1 Hz, with the fifth cycle used for data analysis.^[^ ^137^ ^]^ Disc stiffness was assessed by calculating the gradient of the force‐displacement curves within the “toe” (0.1–0.2 MPa) and “linear” (2.2–2.3 MPa) regions. FSU height was measured before and after loading by bringing the load cell into contact with each FSU and recording the relative height at which 2 N was reached. The loss in disc height was defined as the difference between these measurements. Statistical significance was assessed using mixed effects analysis with the Geisser‐Greenhouse correction plus Tukey's multiple comparisons test, which appropriately handles unequal sample sizes and repeated measures, with individual variances computed for each comparison ^(*^ *p* <0.05).

## Conflict of Interest

V.N., J.P.K.A., M.M.S., M.D.G., and C.C.C. have published a patent (PCT/GB2020/051847) on ultrasound‐triggered liposome payload release. M.M.S. has invested in, consults for (or is on scientific advisory boards or boards of directors), and conducts sponsored research funded by companies related to the biomaterials field, and has co‐founded companies in the biomaterials field. C.C.C. is a founder, director, consultant, shareholder, and receives consultancy income from OrthoSon Ltd, a company commercializing stimulus‐responsive injectable biomaterials for degenerate disc repair and the treatment of lower back pain. It should be noted that there is presently no relationship between the research described in this paper and the products being commercialized by OrthoSon. The rest of the authors declare no conflict of interest.

## Author Contributions

V.A.B. and A.P.C. are co‐first authors. V.A.B. designed and optimized the ultrasound setup and parameters for both the in vitro and ex vivo experiments and developed custom‐written algorithms for the automated control of ultrasound exposure. V.A.B. conducted acoustic attenuation measurements of hydrogels with and without microspheres, as well as bovine tissue, and led the development of sound‐absorbing hydrogels with matched attenuation to bovine tissue through the addition of microspheres. Additionally, V.A.B. prepared and characterized (DLS) the liposomes using the Genizer‐Microfluidizer system without the interdigitation‐fusion method, prepared the bovine IVD material for rheological testing, performed the calcium concentration assay, and the enzymatic degradation of ex vivo IVD. A.P.C. prepared the liposomes using the Avanti Polar mini‐extruder, with and without the interdigitation‐fusion method, and provided the liposomes for all but the cavitation‐guided gelation experiments. A.P.C. also performed the liposome characterization via DLS and the polymer characterization via NMR. In addition, A.P.C. optimized the hydrogel formulation by investigating a variety of hydrogel systems, characterized the injectability of the precursor material, and performed the hydrogel characterization via rheological measurements. A.P.C. also performed the unconfined compression tests on the hydrogels and characterized the bovine NP by rheology. M.J.K. assisted with the ex vivo proof‐of‐concept experiment and performed the compressive testing of treated functional spinal units. V.A.B., A.P.C., and M.J.K. have performed the data analysis on their respective parts, and they have written the relevant parts in the manuscript. Both V.A.B. and A.P.C. contributed to writing the introduction part of the manuscript. V.N. performed the temperature‐induced calcium release experiment, including the liposome preparation, and produced preliminary data that contributed to conceiving this project. D.R. performed the in vitro biocompatibility tests. L.B. assisted with the calcium concentration assay and the optimization of ultrasound treatment algorithms, provided guidance on liposome formulation and production using the Genizer‐Microfluidizer system, and offered ongoing support throughout the project. S.J.P.C. trained A.P.C. on the unconfined compression tests and supervised the relevant data analysis. J.P.K.A. contributed to the grant idea and writing‐up, supervised V.N., and is listed as co‐investigator on the FUSF grant. N.N. is listed as co‐investigator on the FUSF grant and supervised M.J.K. C.C.C. is listed as co‐investigator on the FUSF grant and supervised the project. M.M.S. was listed as co‐investigator on the FUSF grant, and was the project's co‐supervisor and co‐administrator. M.D.G. was the main investigator on the FUSF grant, and was the project's co‐supervisor, co‐administrator, and organizer. All co‐authors had provided feedback on the manuscript, which had been applied by V.A.B., A.P.C., and M.J.K. The manuscript was approved by all authors before submission.

## Supporting information



Supporting Information

## Data Availability

The data that support the findings of this study are available from the corresponding author upon reasonable request.
