# Supplementary material for: Ultrasound‐Triggered Gelation for Restoring Biomechanical Properties of Degenerated Functional Spinal Units
Source: Adv Healthc Mater. 2025 Dec 10;15(7):e01823. doi: 10.1002/adhm.202501823 (PMC12908214; doi:10.1002/adhm.202501823)
Supplement: Supplementary file 1 — Supporting Information [file ADHM-15-0-s001.docx]

Supporting Information


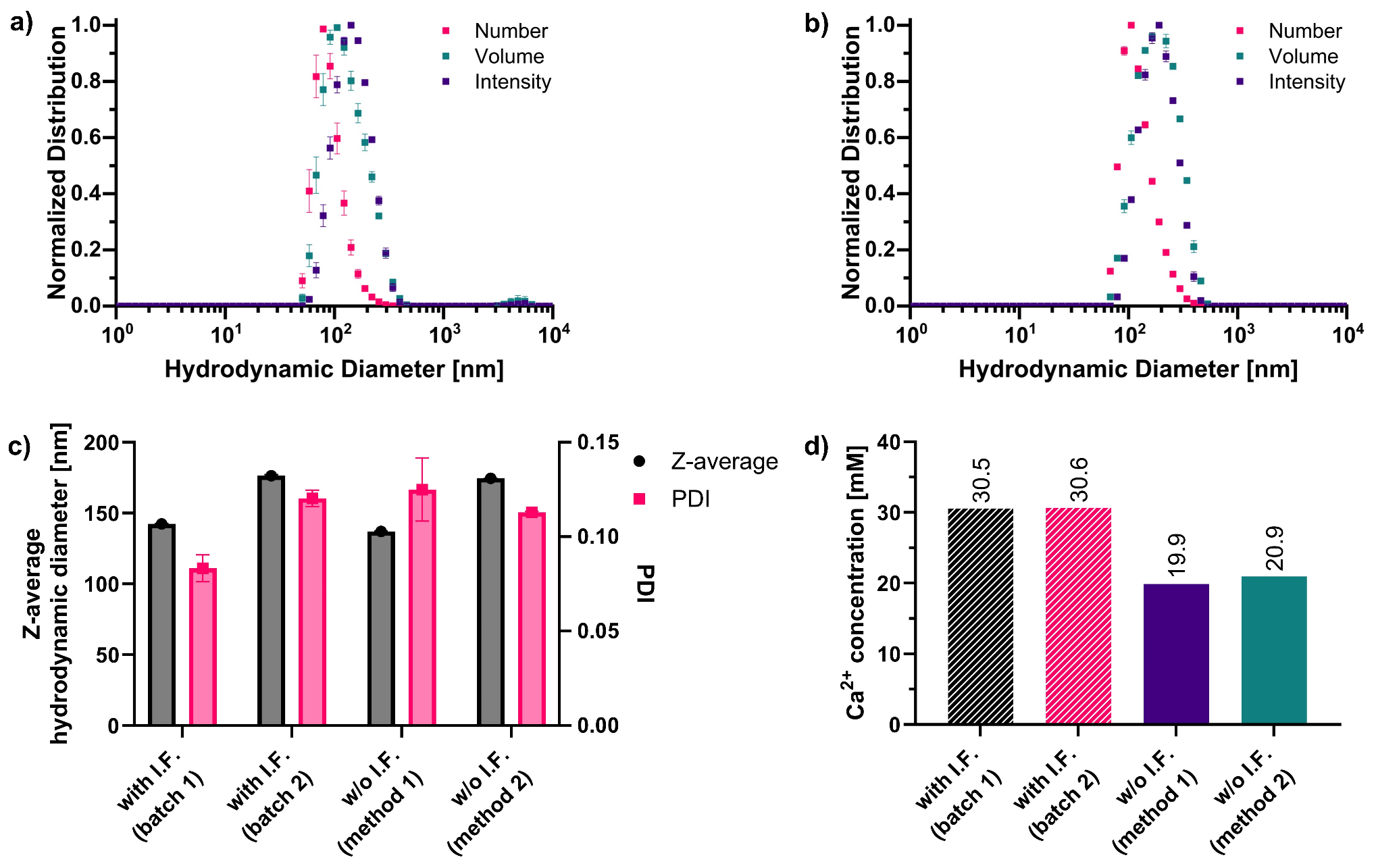


**Figure S1:** a) Normalized number, volume, and intensity distributions for the liposome formulation produced by using the microfluidizer without interdigitation-fusion [w/o I.F. (method 1)] at a concentration of 0.02 mg/mL in iso-osmotic buffer (mean ± S.E. based on n = 3 liposome batches). b) Normalized number, volume, and intensity distributions for the liposome formulation produced by using the mini-extruder without interdigitation-fusion [w/o I.F. (method 2)] at a concentration of 0.02 mg/mL in iso-osmotic buffer (mean ± S.E. based on n = 3 technical repeats). c) *Z*-Average hydrodynamic diameters (black) and PDI values (pink) of liposomes produced via different methods: i) with interdigitation-fusion (with I.F., mean ± S.E. based on n = 3 technical repeats), ii) without interdigitation-fusion (w/o I.F., method 1: mean ± S.E. based on n = 3 liposome batches, and method 2: mean ± S.E. based on n = 3 technical repeats). d) Total calcium concentration in liposomes produced via the different methods detailed in c).


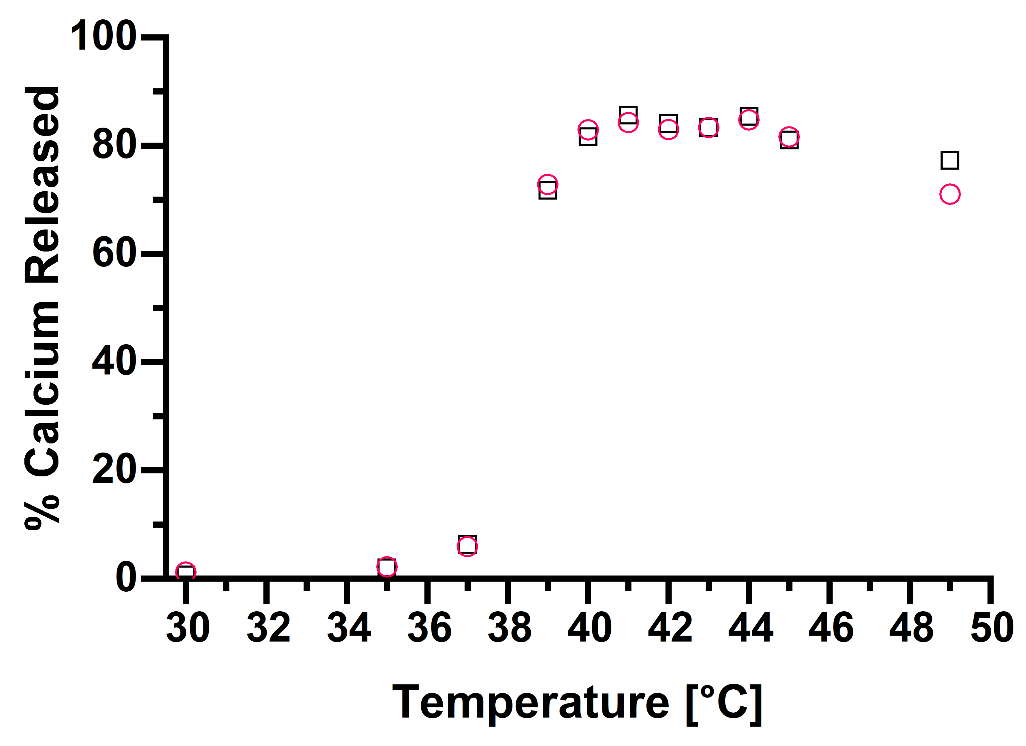


**Figure S2:** Percentage of released calcium from interdigitation-fusion liposomes based on DPPC:DSPE PEG biotin = 99:1 mol ratio as a function of different incubation temperatures for n = 2 technical repeats.


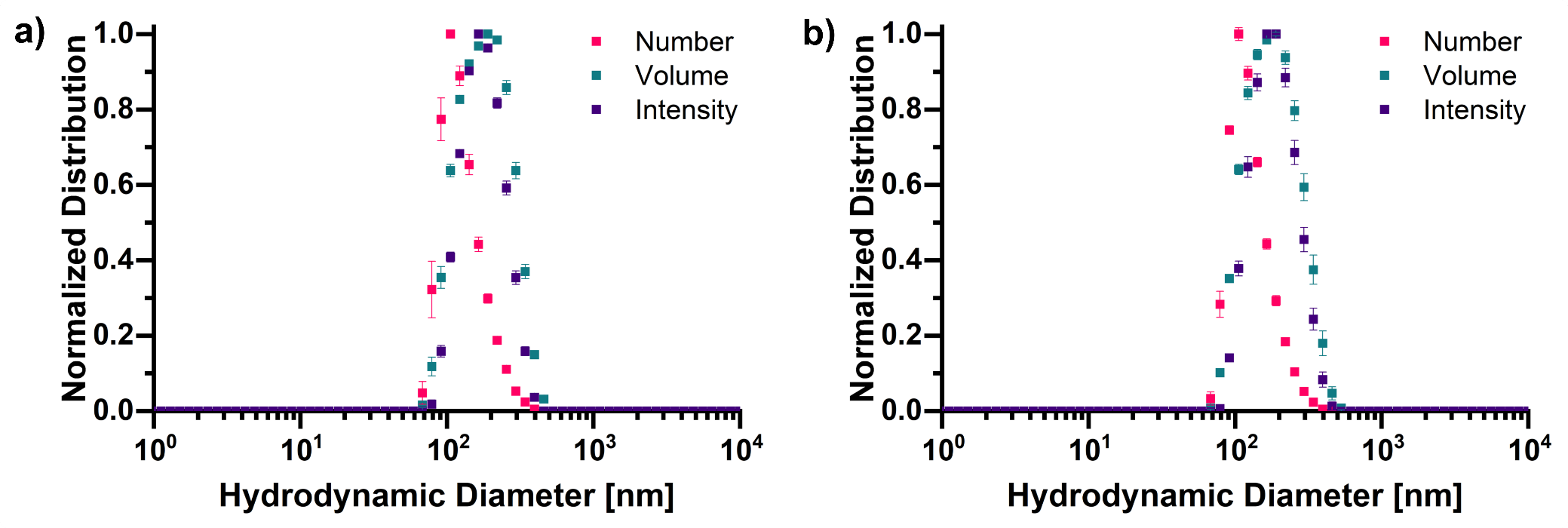
**Figure S3:** a) Normalized volume, number, and intensity distributions for calcium-loaded interdigitation-fusion liposomes based on (DPPC:DSPE PEG = 99:1 mol ratio) at a concentration of 0.5 mg/mL in iso-osmotic buffer, immediately after fabrication (*Z*-Avg = 166.7 ± 0.5 nm, PDI = 0.101 ± 0.008). b) Size distributions at a concentration of 0.5 mg/mL in iso-osmotic buffer 1 month after storage at 2 °C (*Z*-Avg = 173 ± 1 nm, PDI = 0.13 ± 0.01). Data are reported as the mean ± S.E. based on n = 3 technical repeats.


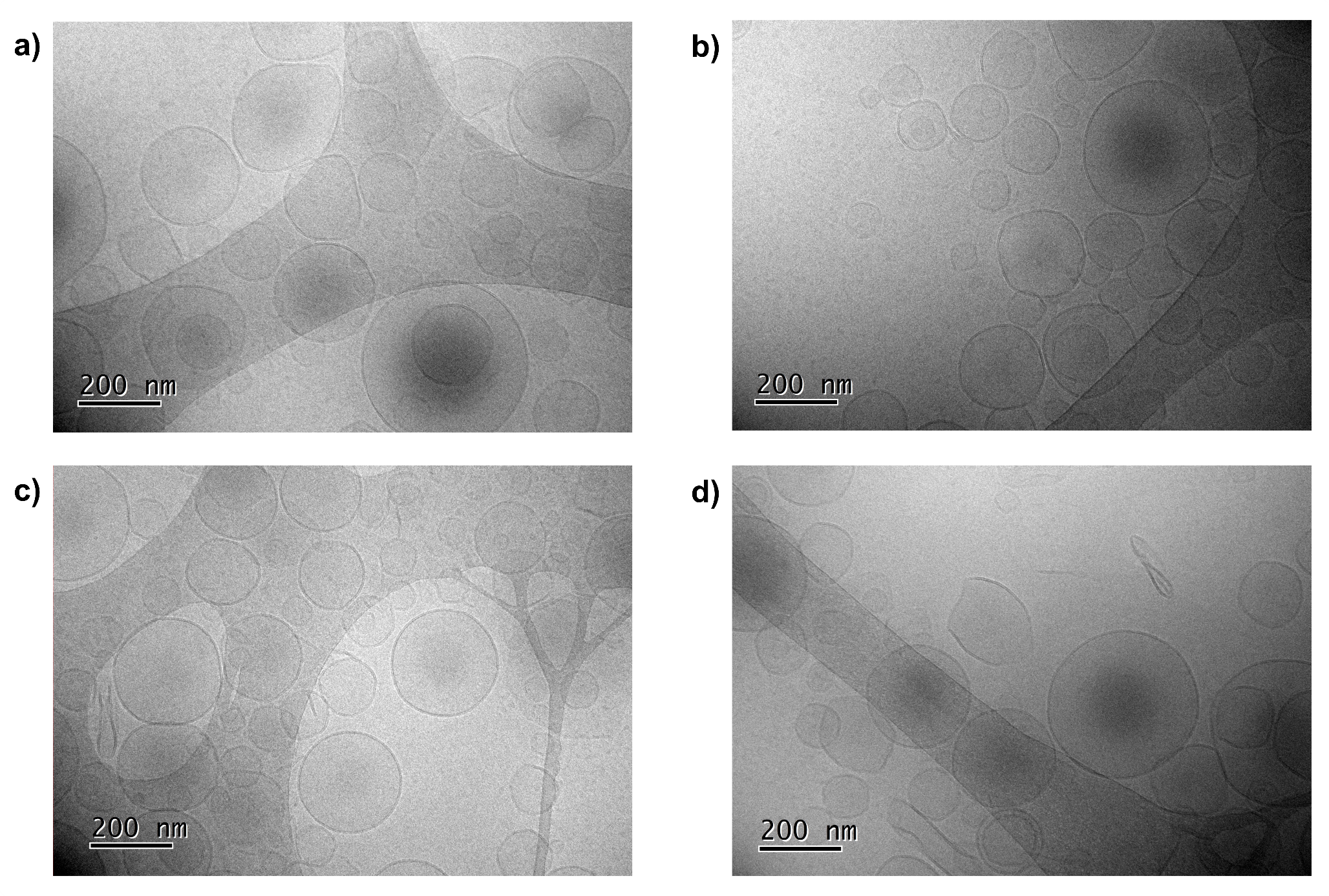


**Figure S4:** CryoTEM images of the liposome formulation at 20 mg/mL in iso-osmotic buffer at 20 °C. The liposomes consist of 99:1 molar ratio DPPC and DSPE-PEG2000 lipids and were produced via the interdigitation-fusion method.

**
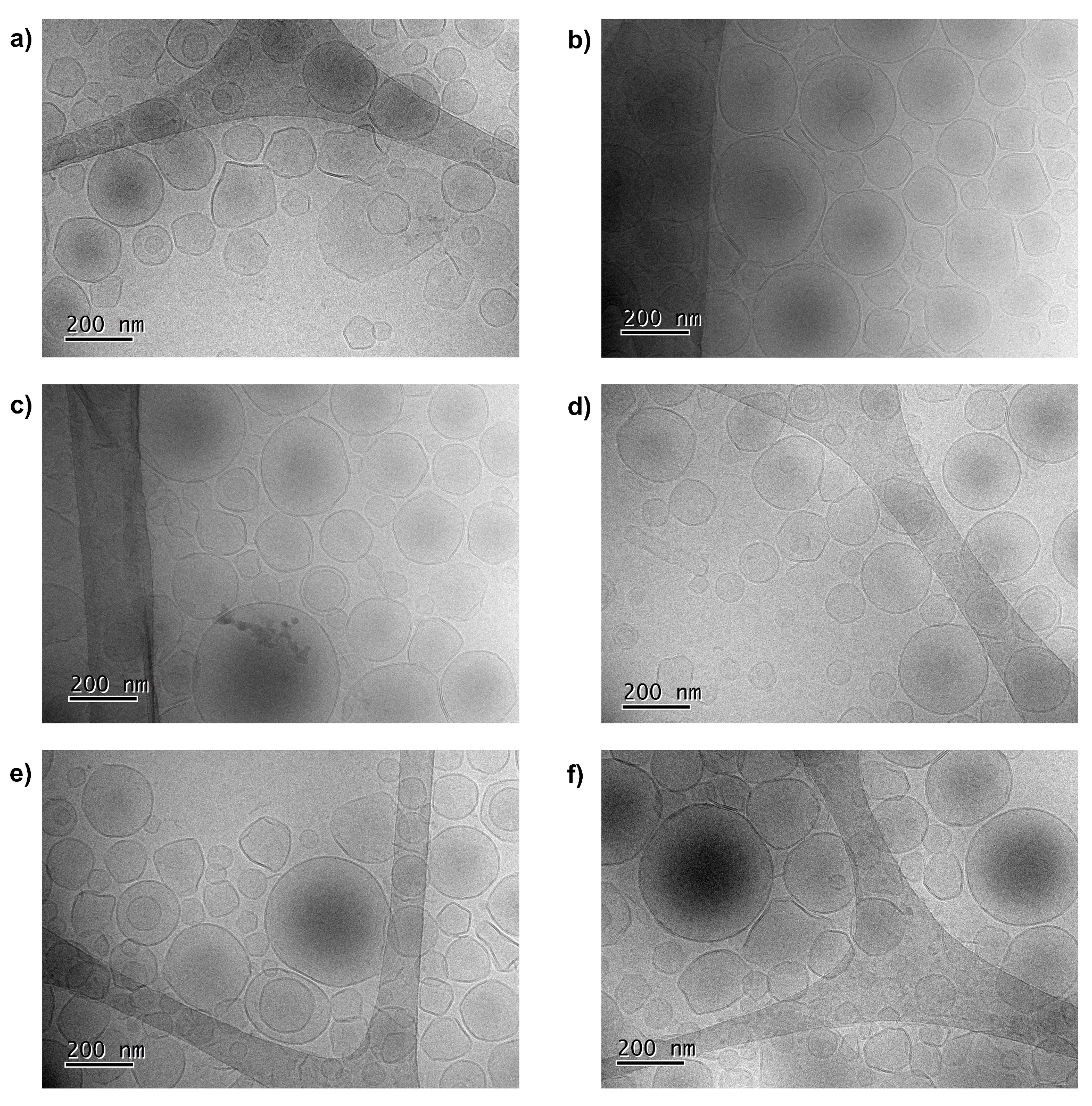
Figure S5:** CryoTEM images of the liposome formulation at 20 mg/mL in iso-osmotic buffer, after being heated to 42 °C. The liposomes consist of 99:1 molar ratio DPPC and DSPE-PEG2000 lipids and were produced via the interdigitation-fusion method.


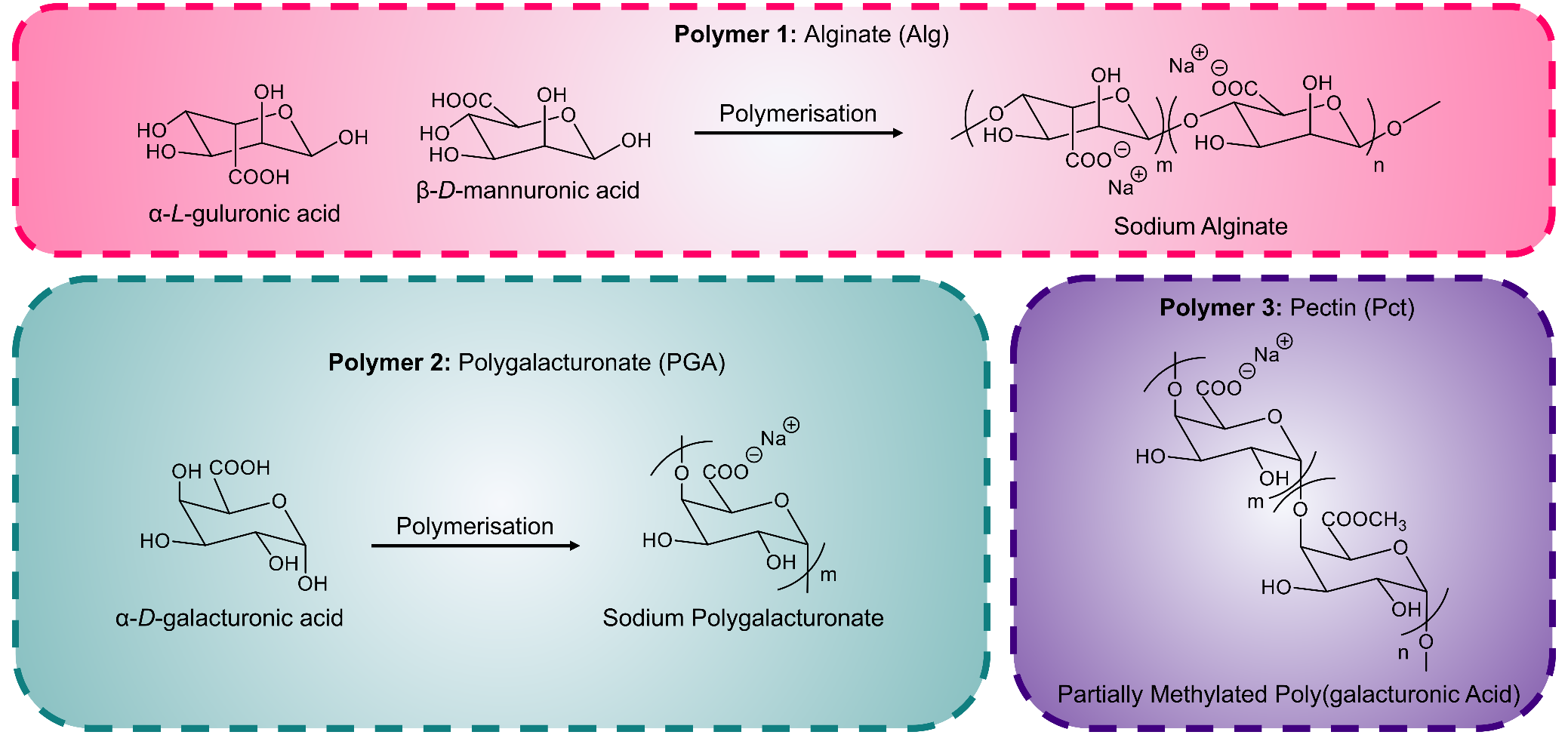


**Figure S6:** Chemical structures of the anionic polysaccharides used in this project. Sodium alginate polymer consists of α, L-guluronic acid, and β-D-mannuronic acid residues, sodium polygalacturonate consists of α, D-galacturonic acid, and pectin mainly consists of the methylated ester of poly(galacturonic acid).


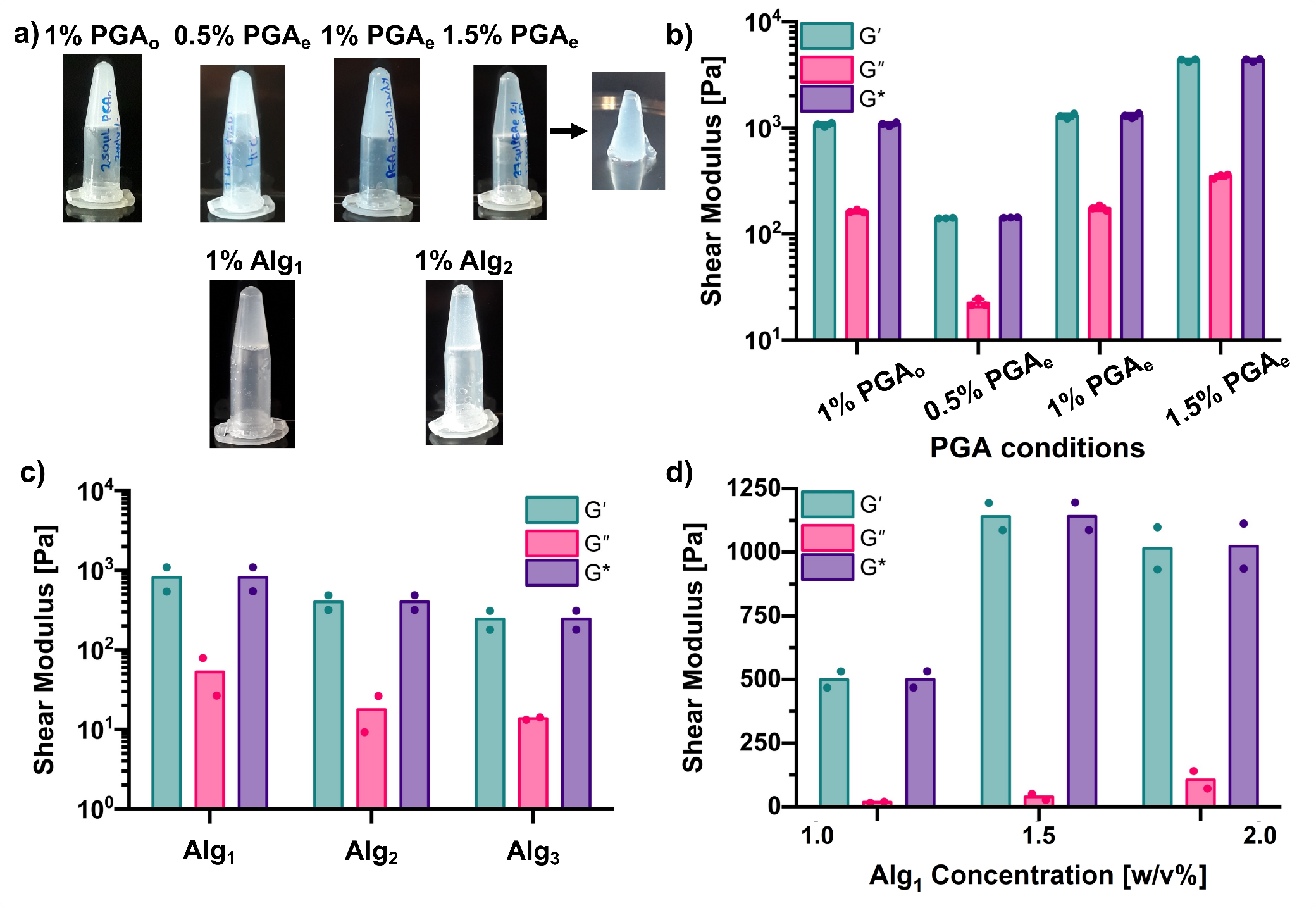
Figure S7: a) Photographs of the formulations containing either PGA or Alg and liposomes, upon heating to 41 °C, followed by tube-inversion. b) Shear moduli of formulations containing PGA_o_ at polysaccharide concentration of 1% w/v, and PGA_e_ at different polysaccharide concentrations (mean ± S.E. based on n = 3 technical repeats). c) Shear moduli values of hydrogels produced using different alginate sources (mean based on n = 2 liposome batches). d) Effect of Alg_1_ concentration on the shear moduli of the hydrogels (mean based on n = 2 liposome batches). The shear modulus values were determined by frequency sweep measurements at 0.1% shear strain and 1 rad s^‒1^ angular frequency. The details of the different polysaccharides tested can be found in the Materials section.

**
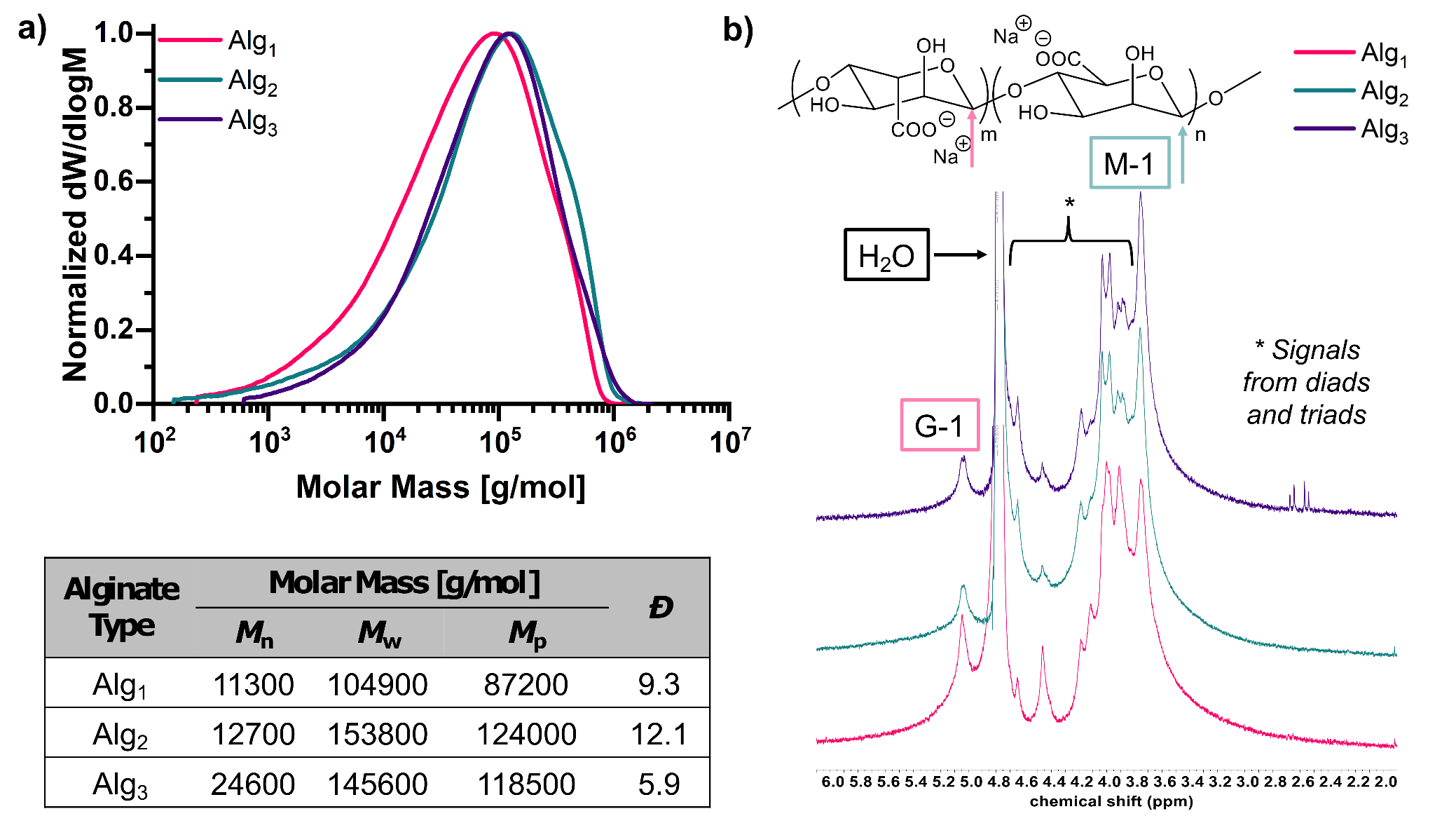
Figure S8:** a) GPC traces of the different alginates in 0.1 M NaNO_3_ aqueous solution, and their molar mass characteristics based on universal calibration, and b) ^1^H NMR spectra of the different alginates in D_2_O.

**
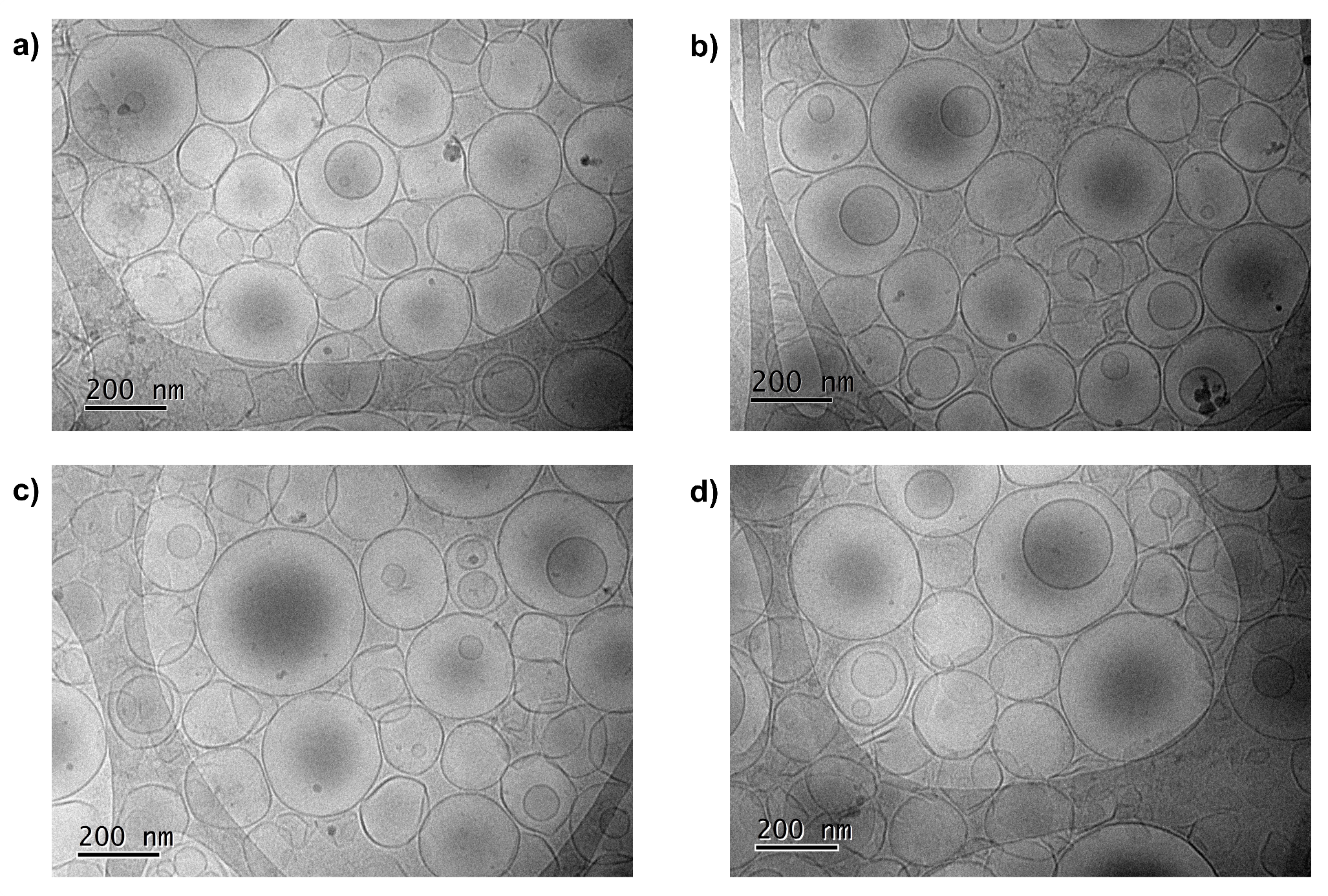
Figure S9:** CryoTEM images of the hydrogel precursor containing 1.5% w/v Alg_1_, 10 mg/mL liposomes loaded with 15 mM calcium chloride, in 0.3 M NaCl at 20 °C. The liposomes consist of 99:1 molar ratio DPPC and DSPE-PEG2000 lipids and were produced via the interdigitation-fusion method.


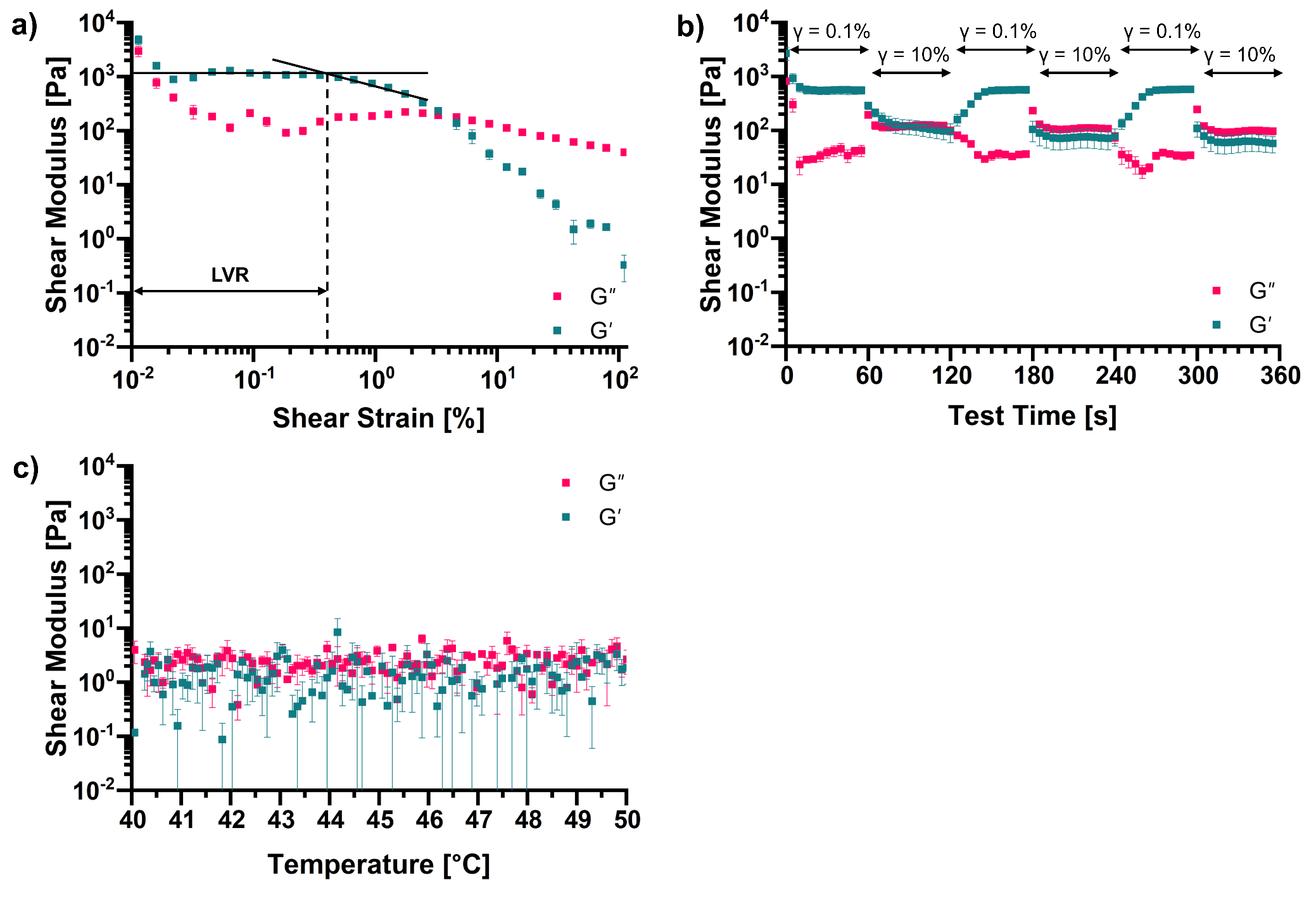
**Figure S10:** a) Amplitude sweep measurements on the hydrogels (1.5% wt/v Alg_1_ and 15 mM calcium-loaded liposomes in 0.3 M NaCl) at 37 °C and angular frequency of 1 rad s^‒1^ indicating the LVR of the samples (mean ± S.E. based on n = 3 liposome batches). b) Strain recovery measurements on the hydrogels (1.5% wt/v Alg_1_ and 15 mM calcium-loaded liposomes in 0.3 M NaCl) at 37 °C (mean ± S.E. based on n = 3 liposome batches). c) Temperature ramp measurement on the Alg_1_ polymer solution (1.5% wt/v) without liposomes, as a control, performed at angular frequency of 1 rad s^‒1^ and shear strain of 0.1% (mean ± S.E. based on n = 3 different samples).


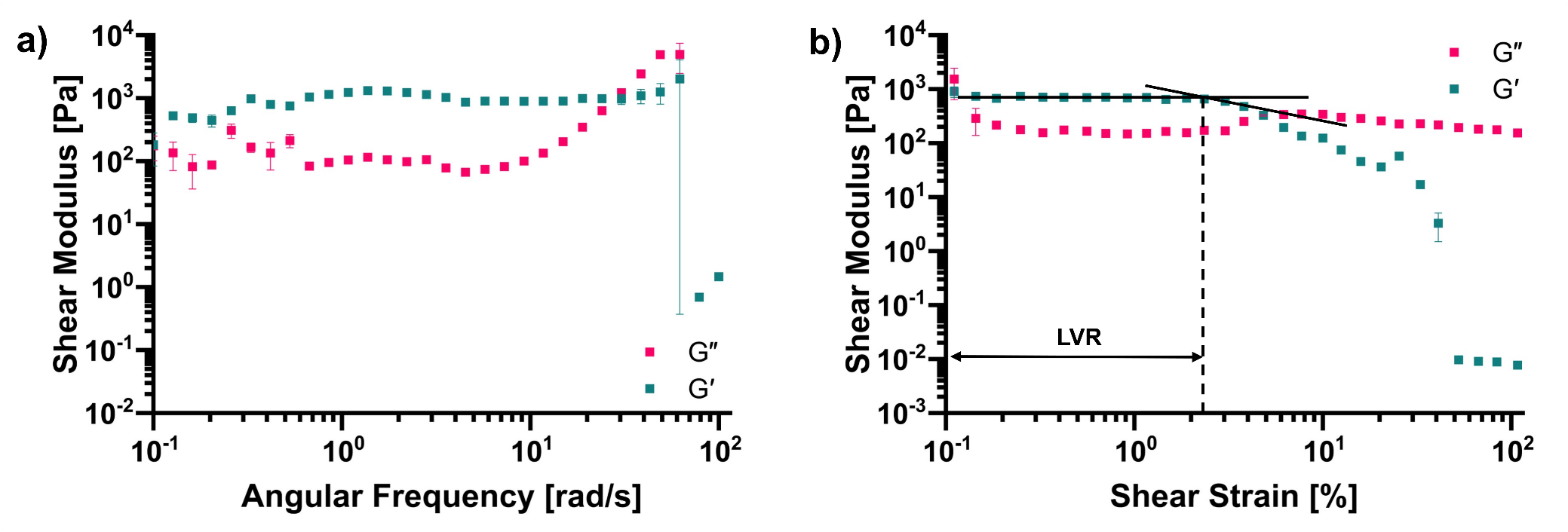


**Figure S11:** Rheological properties of the hydrogels made of 1.5% wt/v Alg_1_ and 15 mM calcium-loaded liposomes in 0.3 M NaCl. These correspond to sample 1.1 in **Figure 5**. The hydrogels were exposed to ultrasound, and they were tested as extracted from the sample holder, without further processing. a) Frequency-sweep measurements on the hydrogels at 37 °C and shear strain 0.5% (mean ± S.E. based on n = 3 technical repeats. b) Amplitude sweep measurements on the hydrogels at 37 °C and angular frequency of 1 rad s^‒1^ indicating the LVR of the samples (mean ± S.E. based on n = 3 technical repeats).

**
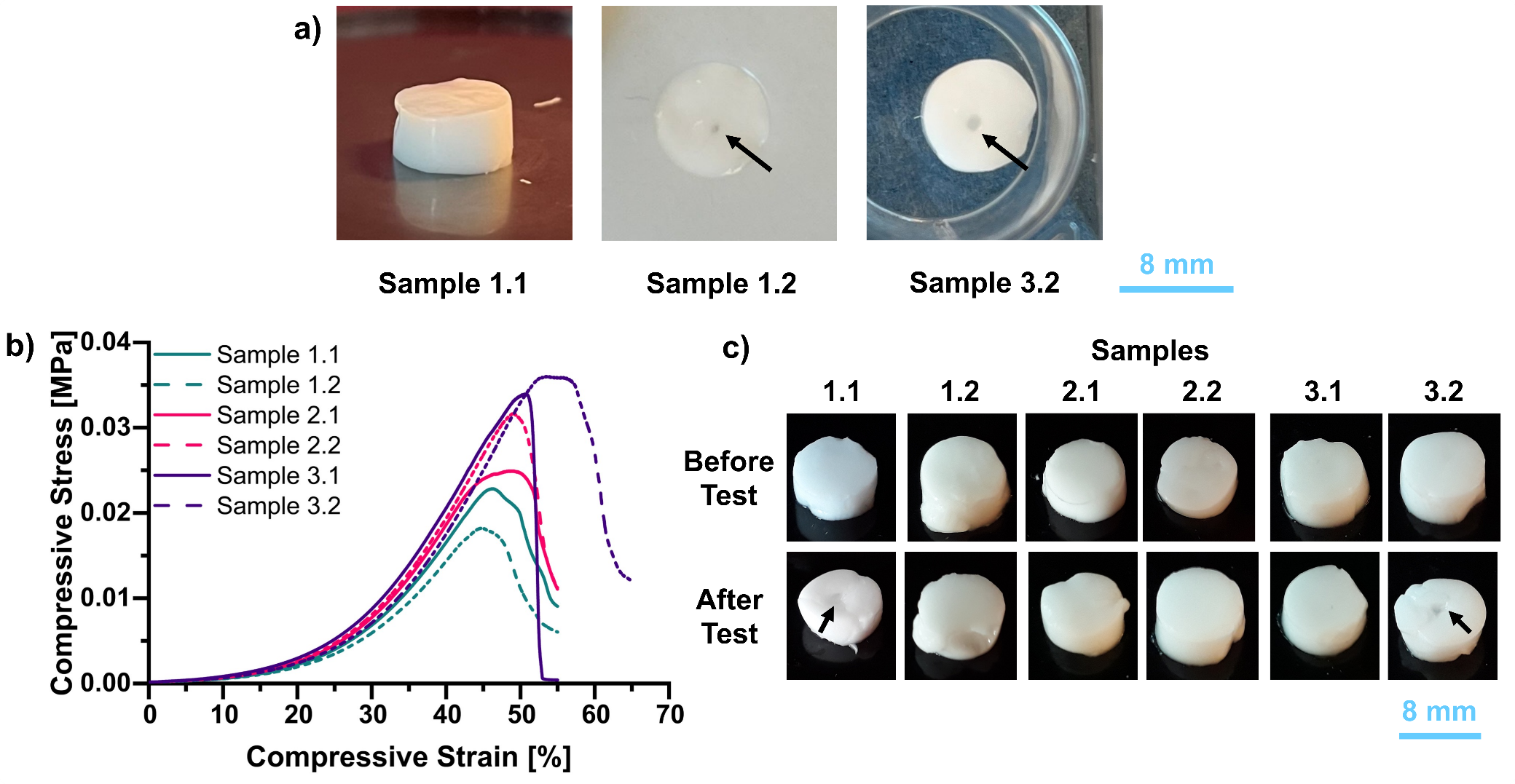
Figure S12:** Samples of 1.5% wt/v Alg_1_ in ~0.3 M NaCl, 6% wt/v microspheres, and thermosensitive liposomes containing 10-15 mM calcium chloride. Three liposome batches were used, and samples are labelled as x.y, where x corresponds to the liposome batch, and y corresponds to the replicates within the same liposome batch. a) Photographs taken after cutting the ultrasonically triggered hydrogels to a height of 4 mm showing the presence of a hole caused by the thermocouple, b) Compressive stress as a function of compressive strain, as a result of the unconfined compression test on ultrasonically-triggered hydrogels, and c) Photographs of the hydrogels before and after compression test, indicating that the hole acted as initiation point for failure. Scale bar set at 8 mm.


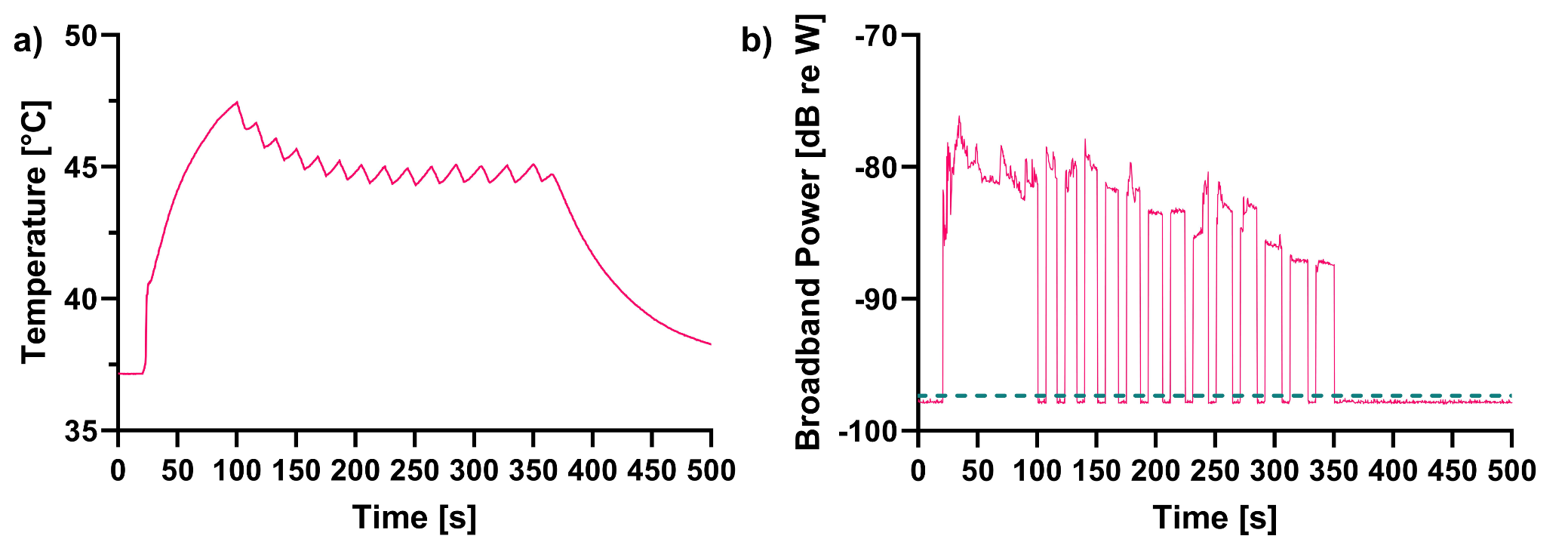


**Figure S13:** Heating curve (a) and PCD broadband energy data (b) for Sample-US (1.5% wt/v low-viscosity alginate in ~0.3 M NaCl, 6% wt/v microspheres) produced using cavitation-guided gelation for comparison to the incubator-treated and bovine NP sample in **Figure 6h**.


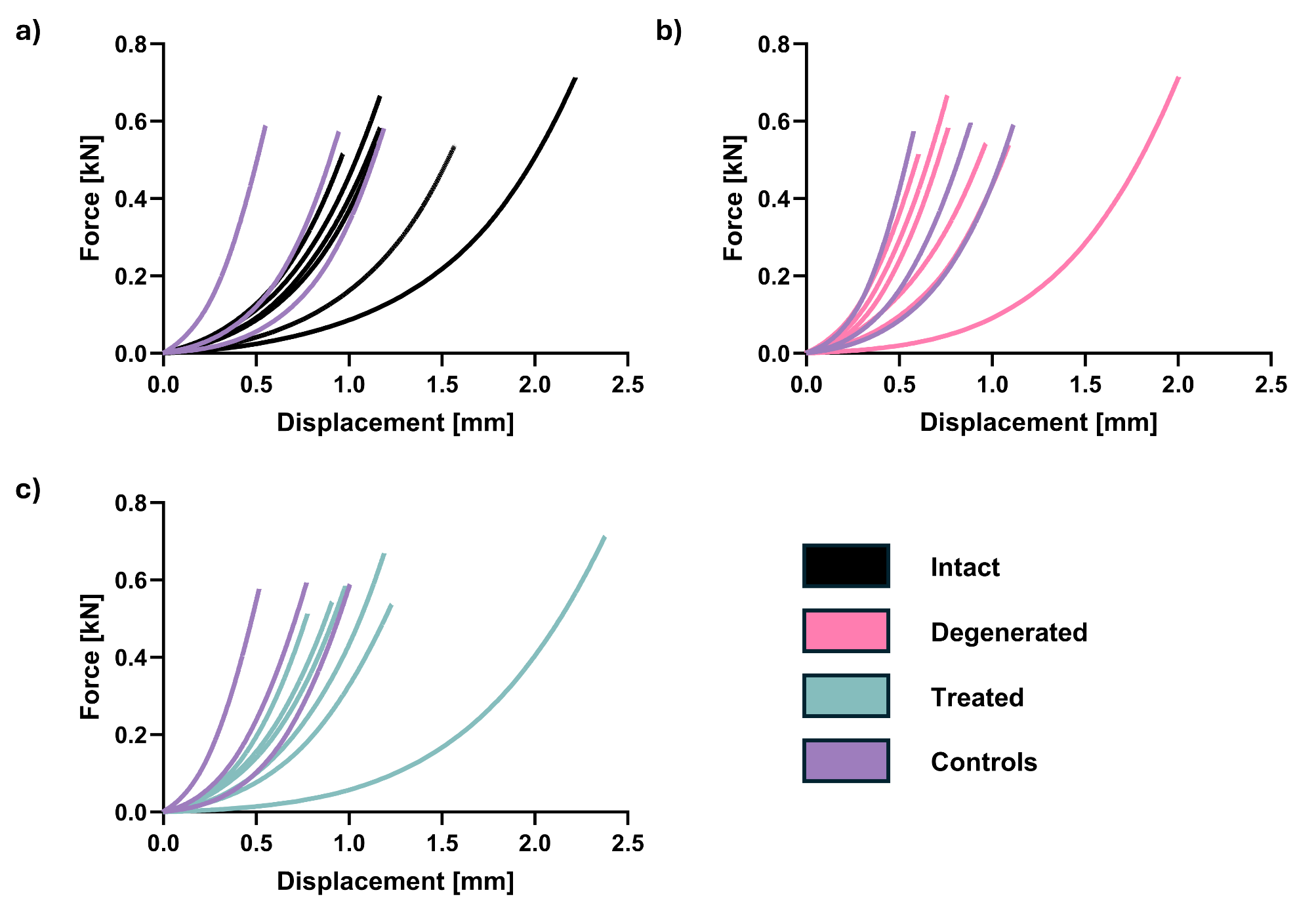


**Figure S14**: Biomechanical assessment of functional spinal unit performance under uniaxial compression when a) intact, b) following degeneration, and c) following treatment, compared against controls.

**
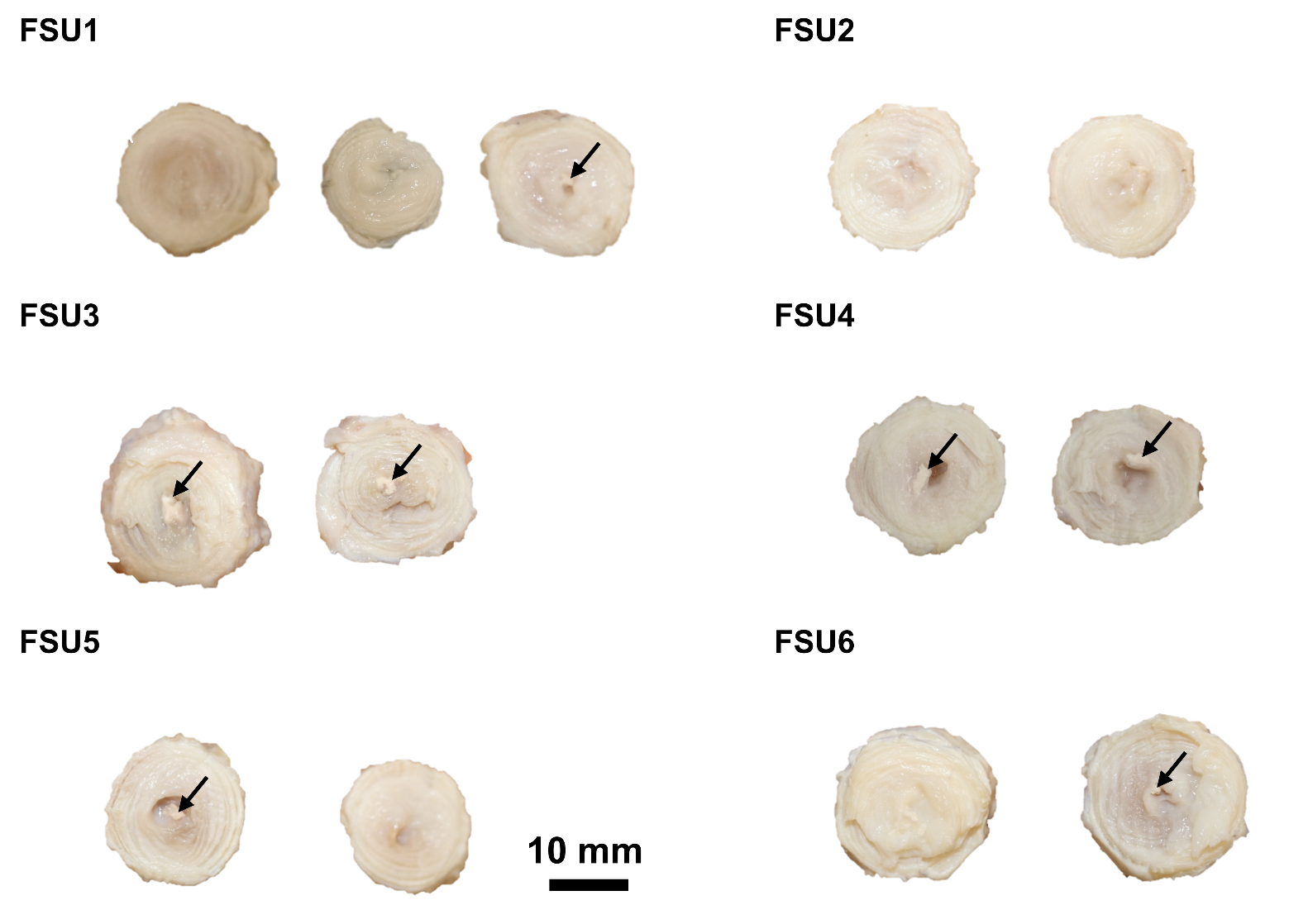
**

**Figure S15:** Axial cross-sections of the six treated FSU specimens showing the integration of the crosslinked hydrogels (indicated by black arrows) within the nucleus pulposus (NP) immediately after biomechanical testing. FSU 1 was sectioned at three different levels to provide a clearer view of gel integration.

**Table S1**. Components of the Attenuation Measurement Experiments

| Item | Manufacturer |
| --- | --- |
| Focused source | Olympus NDT V311, 10 MHz center frequency, 0.5" diameter, 7.6 cm geometric focus, Waltham, Massachusetts, USA |
| Pulser | DPR300, JSR Ultrasonics, Pittsford, New York, USA |
| Hydrophone | Precision Acoustics NH0200, Dorchester, UK |
| Sample Holder | 10 mm inner diameter, 8 mm interior thickness, custom design made from clear Perspex |

**Table S2:** Dimensions and compressive test conditions of the functional spinal units.

| **Sample** | **Group** | **Cross-sectional area**  **[mm^2^]** | **Loading range**  **[MPa]** | **Maximum load**  **[N]** | **Loading rate**  **[Hz]** | **Loading rate**  **[N s^-1^]** |
| --- | --- | --- | --- | --- | --- | --- |
| FSU1 | Treatment | 337.4 | 0.1-2.3 | 512.2 | 1.0 | 974.4 |
| FSU2 | Treatment | 341.1 | 0.1-2.3 | 517.8 | 1.0 | 985.6 |
| FSU3 | Treatment | 420.4 | 0.1-2.3 | 638.2 | 1.0 | 1226.4 |
| FSU4 | Treatment | 342.7 | 0.1-2.3 | 492.9 | 1.0 | 935.9 |
| FSU5 | Treatment | 368.8 | 0.1-2.3 | 559.8 | 1.0 | 1069.6 |
| FSU6 | Treatment | 445.8 | 0.1-2.3 | 676.8 | 1.0 | 1303.5 |
| FSU7 | Control | 371.9 | 0.1-2.3 | 564.5 | 1.0 | 1079.0 |
| FSU8 | Control | 372.7 | 0.1-2.3 | 565.7 | 1.0 | 1081.5 |
| FSU9 | Control | 362.5 | 0.1-2.3 | 550.3 | 1.0 | 1050.7 |
